# Complete inhibition of a polyol nucleation by a micromolar biopolymer additive

**DOI:** 10.1016/j.xcrp.2021.100723

**Published:** 2022-01-11

**Authors:** Xin Wen, Sen Wang, Robert Ramji, Luke O. Butler, Yelena Bagdagulyan, Audrey Kishishita, James A. Golen, Arnold L. Rheingold, Soo-Kyung Kim, William A. Goddard, Tod A. Pascal

**Affiliations:** 1 Department of Chemistry and Biochemistry, California State University, Los Angeles, Los Angeles, CA 90032, USA; 2 ATLAS Materials Physics Laboratory, Department of NanoEngineering and Chemical Engineering, University of California, San Diego, La Jolla, CA 92093, USA; 3 University of California San Diego Materials Research Science and Engineering Center, University of California, San Diego, La Jolla, CA 92093, USA; 4 Department of Chemistry and Biochemistry, University of California, San Diego, La Jolla, CA 92093, USA; 5 Materials and Process Simulation Center, California Institute of Technology, Pasadena, CA 91125, USA; 6 Lead contact; 7 Present address: Department of Chemistry, California State University, Dominguez Hills, Carson, CA 90747, USA

## Abstract

Preventing spontaneous crystallization of supersaturated solutions by additives is of critical interest to successful process design and implementation, with numerous applications in chemical, pharmaceutical, medical, pigment, and food industries, but challenges remain in laboratory and industry settings and fundamental understanding is lacking. When copresented with antifreeze proteins (AFPs), otherwise spontaneously crystallizing osmolytes are maintained at high supersaturations for months in over-wintering organisms. Thus, we here explore the inhibition phenomenon by AFPs, using persistent crystallization of a common sugar alcohol, D-mannitol, as a case study. We report experimentally that DAFP1, an insect AFP, completely inhibits D-mannitol nucleation. Computer simulations reveal a new mechanism for crystallization inhibition where the population of the crystal-forming conformers are selectively bound and randomized in solution by hydrogen bonding to the protein surface. These results highlight the advantages of using natural polymers to address crystallization inhibition challenges and suggest new strategies in controlling the nucleation processes.

## Graphical Abstract

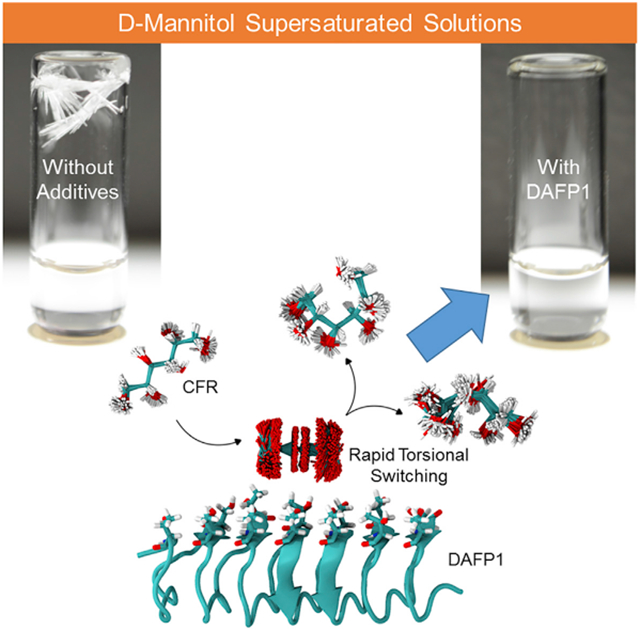

Preventing spontaneous crystallization of supersaturated solutions is important in industrial applications but remains a challenge with limited understanding. Wen et al. show that an insect antifreeze protein completely inhibits D-mannitol nucleation. Computer simulations reveal a dramatic reduction in the crystal forming rotamer population in solution mediated by the protein.

## INTRODUCTION

Crystallization from solution is a ubiquitous phenomenon in nature and in industrial processes, making prevention of spontaneous crystallization of supersaturated solutions of critical interest for successful process design and implementation in various applications including chemical, pharmaceutical, medical, pigment, and food industries. Certain additives, such as tailor-made molecules and complexation agents,^1-3^ have been reported to inhibit or slow down crystallization from supersaturated solutions. However, selection of such additives is a trial-and-error process, with the control often far from optimum and the concentrations required for the additives generally high (far above the micromolar level). Can spontaneous crystallization be completely suppressed from highly supersaturated solutions by a minute amount of additives over months at the nucleation stage? If such an additive exists, how can we find it and how would it function?

**Figure 1.**
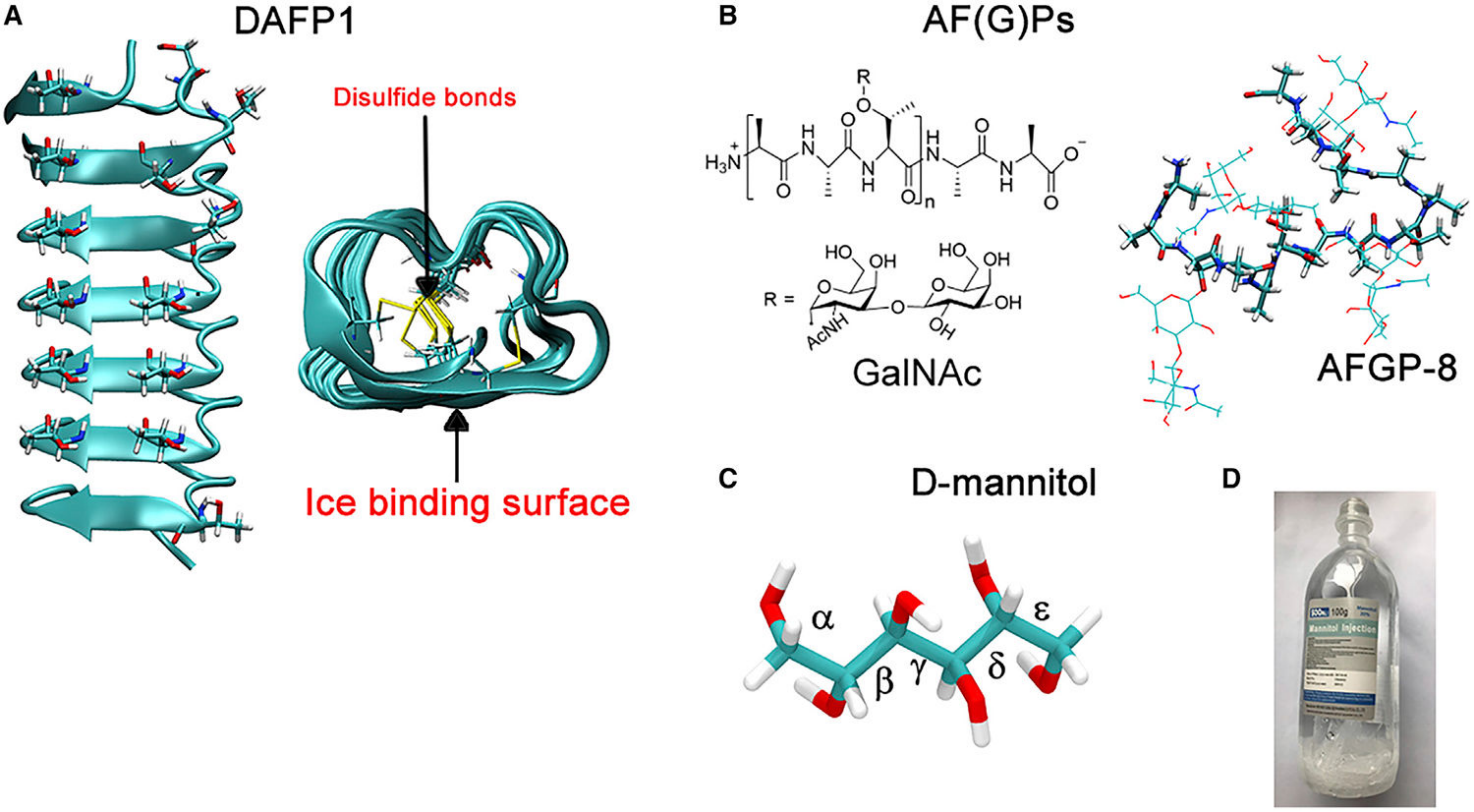
AF(G)P molecular structures (A) Molecular model of DAFP1, where threonine and serine residues on the ice-binding sites (IBS) are shown as licorice. The intra-molecular di-sulfide bonds (cleaved in the denatured structure) are shown in the right inset, where the IBS is also indicated. (B) Structure of AFGPs, where n = 4–55 and R = GalNAc. An atomistic representation in the right inset, where the amino-acid backbone is in licorice, and the GalNAc is shown in ball and stick. (C) Molecular structures of D-mannitol, a stick-ellipsoid representation, showing each of the five O-C-C-O torsions (labeled α, β, γ, δ, and ε, respectively). (D) Commercially available D-mannitol 20% injection solution with substantial D-mannitol crystals at the bottom of the bottle (the picture was shown on the company website).

Nature has evolved ways to tackle this problem strategically. Antifreeze proteins (AFPs) evolved in many cold-adapted organisms (e.g., fishes, insects, plants, bacteria, and fungi) are known to have an extraordinary ability to suppress ice growth through binding to specific ice crystal surfaces.^4-7^ Beetle AFPs usually have well-defined structures and are much more active than their counterparts in fish. For example, a hyperactive beetle AFP from *Dendroides canadensis* (DAFP1) is a 9 kDa β-helical protein with multiple 12- or 13-mer repeats including T-X-T (where X is any amino acid) and eight disulfide bonds with both its overall structure and ice-binding sites (IBSs) well-defined (Figure 1A).^6^ In contrast, fish antifreeze glycoproteins (AFGPs) are intrinsically disordered and contain multiple repeats of the tripeptide A-A-T with the disaccharide β-D-galactosyl-(1→3)-N-acetyl-α-D-galactosamine (GalNAc) that are joined to the threonine residue through a glycosidic linkage (Figure 1 B).^8,9^ Even more remarkably, AFPs have been shown to effectively control precipitation out of solution of certain non-ice like, low-molecular weight organic compounds, such as nucleosides,^10^ monosaccharides,^11^ and disaccharides.^6^ These results imply additional functions for AFPs in cold-adapted organisms especially since AFPs coexist with high concentrations of osmolytes accumulated in cold-adapted organisms for survival of these organisms over the winter, where spontaneously crystallizing osmolytes can be maintained at high supersaturations without crystallization for months.^4-7^ Serving as an important class of osmolytes, sugar alcohols (or polyols) can enhance the antifreeze activity of AFPs.^12^ However, the role of AFPs on sugar alcohols is unknown.

Here, we choose D-mannitol [(2R,3R,4R,5R)-hexane-1,2,3,4,5,6-hexol] (Figure 1C) as a model polyol for several reasons. First, D-mannitol acts as a common cryoprotectant in cold-adapted organisms and is a common polyol in winter-hardy insects.^13^ Furthermore, D-mannitol is the only common sugar alcohol that crystallizes spontaneously from solution.^7^ Moreover, D-mannitol has many uses in a wide range of industries where control of its crystallization is critical. For example, because of its low glycemic index (GI) and safety for teeth, D-mannitol is utilized as reduced-sugar or sugar-free reformulations in the food industry.^14^ D-mannitol is also a commonly used osmotic diuretic treatment to manage fluid build-up conditions (e.g., cerebral edema, increased intracranial pressure) in the medical industry.^15^ In addition, D-mannitol is a popular excipient in solid formulations to stabilize active pharmaceutical ingredients in the pharmaceutical industry.^16^

Many uses of D-mannitol require the compound to remain in its aqueous solution without crystallization. In particular, as a parenteral obligatory osmotic diuretic, the crystallization of D-mannitol injection solution (approximately 15%–25% w/v) must be prevented.^17^ However, D-mannitol solutions readily crystallize during regular storage and manufacturing (Figure 1D), in particular, when exposed to low temperatures. The crystallization of D-mannitol at lower temperatures limits its application as a key formulation excipient (approximately 10%–15% w/v) for frozen or freeze-dried protein drugs and mRNA vaccines.^18,19^ The identification of an effective inhibitor for D-mannitol nucleation is thus imperative for its many industrial uses, but little progress has been achieved so far.

In this work, we show that DAFP1 is an extremely efficient inhibitor of D-mannitol nucleation at highly supersaturated conditions, with complete inhibition observed experimentally over 3 years using only micromolar levels of DAFP1 additive. Based on extensive computer simulations and free energy analysis, we discovered a new mechanism that explains this inhibition, predicting that DAFP1 reduces the population of the crystal forming rotamer by three orders of magnitude, which we predict would extend the crystal induction time to 75 years.

## RESULTS AND DISCUSSION

### Crystallization of D-mannitol

There are several different crystalline forms of D-mannitol, but slow evaporation of D-mannitol aqueous solution yields pure β-form D-mannitol, the most stable form.^20^ Unfortunately, this also often results in mixtures of small crystals that are not suitable for accurate induction time determination. To solve this problem, we tested many crystallization conditions of D-mannitol and found that storing the supersaturated solutions of D-mannitol at 4°C (indeed a common storage mistake for commercially available D-mannitol solutions) yields high-quality pure β-form D-mannitol crystals reproducibly with respect to the induction time and the sizes and shapes of the resulting crystals. This condition was used throughout this study.

Next, we studied the inhibition of D-mannitol crystallization using DAFP1 (Figure 1A), a beetle AFP from *Dendroides canadensis.*^6^ Fish AFGP4-5, a mixture of AFGP4 (18 kDa) and AFGP5 (11 kDa), as well as fish AFGP8 (n = 4), about 2.7 kDa, from *Trematomus borchgrevinki*^9^ were used as AFP controls. Additionally, two other controls, denatured DAFP1 and GalNAc (the disaccharide moiety of AFGPs), were used in this study. All AFPs samples were applied at milli- and micro-molar concentrations.

### Effects of AFGPs and other controls on D-mannitol crystallization

**Figure 2.**
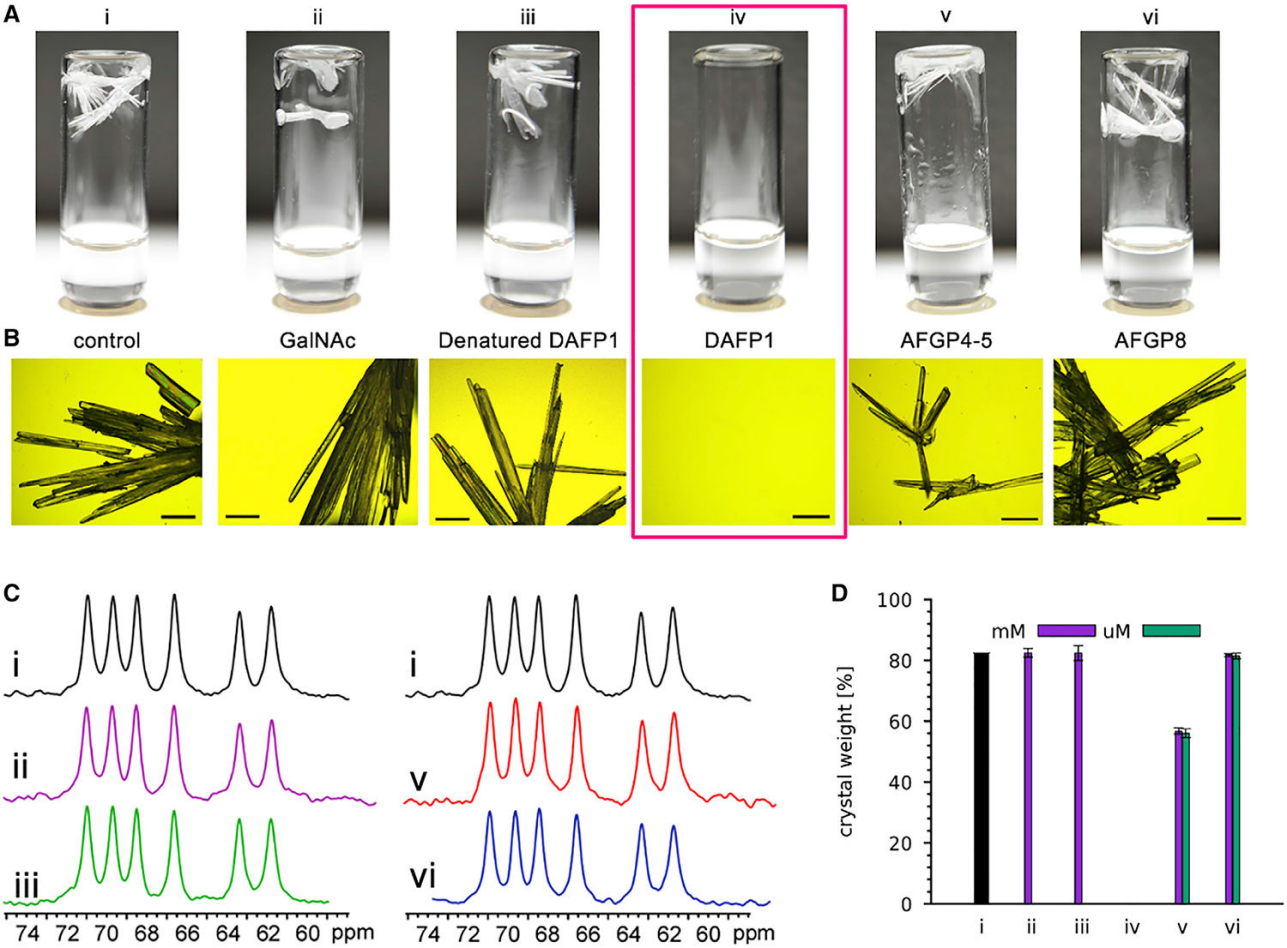
Experimental data for D-Mannitol crystal inhibition by various additives (A) D-Mannitol crystals grown in the absence and presence of additives in vials (shown upside-down), pictured after at least 200 days. Note no crystals form in iv with 1 micromole of DAFP1. (B) Representative optical micrographs of the obtained crystals. The scale bar represents the length of 2 mm. The ordering is the same as in (A). Note again no crystals form in iv with 1 micromole of DAFP1. (C) The CP-MAS ^13^C NMR spectra of finally achieved D-mannitol solids, using the same ordering as in (A). Since the DAFP1 sample has no solids, NMR spectrum of “i,” D-mannitol alone, is re-used here for comparison. (D) Comparison of D-mannitol crystal growth in the absence and presence of additives at milli- (purple) and micro-molar (green) concentrations. Micromolar concentrations of GalNAc and the denatured DAFP1 led to crystals similar to the millimolar concentrations and are omitted. Error bars are for standard deviations.

In the absence of any additives, the first appearance of precipitates from D-mannitol supersaturated solution occurred on day 14 (Table S1). The D-mannitol crystals grown from its supersaturated solution appeared as elongated rods along the *c* axis (Figures 2A and 2B), which is a characteristic shape of β-form D-mannitol. The addition of either control (GalNAc or denatured DAFP1) did not affect the crystallization of D-mannitol: we observed the same induction time, appearance, and weights of the finally achieved D-mannitol crystals from these samples in the presence of the controls. The addition of AFGPs affected D-mannitol crystallization from its supersaturated solution to varying degrees. The presence of 1.0 × 10^−2^ molar AFGP4-5 or AFGP8 delayed the first appearance of D-mannitol precipitates by 49 or 27 days, leading to less amounts of the final crystals at the stop time (Table S1). The crystal habits remained the same, although they appeared as relatively smaller elongated rods. The inhibitory effect of AFGP4-5 on D-mannitol crystallization was more significant than that of AFGP8, in that higher concentrations of AFGP4-5 had a more pronounced effect in delaying the induction time of D-mannitol crystallization. Such concentration effects were not obvious for AFGP8. For effective inhibition of crystallization, interactions between the additive and the compound of interest are generally required.^3^ The results of AFGP8 are more similar to those of the negative controls, indicating that AFGP8 has low affinity to D-mannitol. These results also suggest that the crystallization of D-mannitol can only be affected by AFGPs to a very limited extent, and which may be through weak suppression of the crystal growth of D-mannitol.

The final crystals were all checked using single crystal X-ray diffraction and confirmed to be β-form D-mannitol. Specifically, the crystallographic data (Table S2) of D-mannitol crystals achieved in the presence of denatured DAFP1 were analyzed and deposited in the Cambridge Crystallographic Data Center (CCDC number: 2015391). These results are in good accordance with those published previously^20^ and were used for the structural analysis herein (Table S2 and the supplemental experimental procedures).

To characterize subtle differences in the hydrogen bonding (HB) arrangements of the final D-mannitol solids, we utilized ^13^C cross-polarization magic angle spinning (CP-MAS) NMR spectroscopy (Figure 2C) and attenuated total reflectance Fourier-transform infrared (ATR-FTIR; Figure S1 and the supplemental experimental procedures) spectroscopy. The spectra of the D-mannitol solids achieved in the presence of the AFGPs and the controls were identical to those of the D-mannitol solids obtained in the absence of additives, which are in good agreement with published data for β-form D-mannitol,^21^ indicating that all crystallites are pure β-form D-mannitol. Thus, it is possible that AFGPs may to some extent suppress the continued crystal growth of D-mannitol, but there is no apparent adsorption of AFGPs on the crystal surfaces of D-mannitol.

### Complete inhibition of D-mannitol nucleation by micromolar DAFP1

In contrast to all the controls including AFGPs, DAFP1 showed a dramatic inhibition of nucleation of D-mannitol from its highly supersaturated aqueous solution ( Figure 2D). Notably, potential effects of DAFP1 on the supersaturation degree of D-mannitol have been ruled out by the control experiments (Table S1) and then confirmed by the seeding experiments (Figure S2 and the supplemental experimental procedures). Moreover, Figure S2 shows that DAFP1 can effectively reduce the growth speed of the D-mannitol crystals with an inhibitory effect that is much more significant than that of AFGPs. This suggests that when seed crystals are present, DAFP1 can also reduce the rate of crystal growth, slowing down crystallization of D-mannitol. Indeed, no crystallization of D-mannitol in the presence of DAFP1 has been observed after more than 3 years’ storage despite the lower, micro-molar (2.8 × 10^−6^ M) concentration. Furthermore, the structural integrity of D-mannitol was assessed by NMR spectroscopy. ^1^H and ^13^C NMR spectra (Figures S3 and S4 and the supplemental experimental procedures) confirm the integrity of the 3-year-old D-mannitol structure, which rules out the possibility that the lack of crystallization was due to the loss of the structural integrity of D-mannitol in the solution. Thus, this dramatic suppression of nucleation is unprecedented in terms of the level of the additive used and the duration.

D-mannitol solutions can be highly supersaturated by cooling,^15,22^ and the metastable zone width (MSZW) of D-mannitol is narrow across a wide temperature range^14^ suggesting that the 1 M D-mannitol at 4°C exceeds the critical supersaturation of D-mannitol for nucleation. Indeed, we observed D-mannitol crystallization in 2 weeks in the absence of additives (Figure 2). How can micromolar DAFP1 suppress the nucleation of highly supersaturated 1 M D-mannitol solutions for over 3 years? Inhibition by adsorption,^1,23^ the generally accepted mechanism for crystallization inhibition, cannot explain this phenomenon because there are no crystal growth for D-mannitol in the presence of DAFP1. Further, at 1 M concentration, our D-mannitol solutions at 4°C are far above the critical saturation point, which rules out the possibility that the observed inhibition arises from enhanced solubility due to the addition of DAFP1 at a micromolar level. Thus, we postulated that DAFP1 must somehow reduce dramatically the concentration of the key conformation required for forming the nucleus and a major conformer with a dimer network on the fastest growth faces of the D-mannitol crystal is the key crystal forming rotamer (CFR).

### Stable D-mannitol conformers and their interactions with DAFP-1 *in silico*

In order to discover the atomistic mechanism underlying the nucleation inhibition of D-mannitol by DAFP1, we carried out a series of molecular dynamics (MD) simulations. The chiral D-mannitol molecule has 5 C – C backbone bonds, leading to 3^5^ = 243 possible stable conformations of the O-C-C-O torsional angles (Table S3), where the torsional angles are near trans (t = 180°), positive gauche (p = −60°) or negative gauche (n = −60°). Free energy analysis reveals that the T-T-P-T-T rotamer (BR) is the most favorable in the bulk solution at 277K, comprising 33.7% of the population. In contrast, the crystal forming rotamer (CFR) is N-T-P-T-N with just a 0.45% population. There is typically a large barrier for torsional rotation in the bulk solution, which precludes rapid conformational switching (Figure S5). Indeed, accelerated MD simulations^24,25^ show barriers for internal β and δ torsional rotations of ~80 kJ/mol (Figure S6).

**Figure 3.**
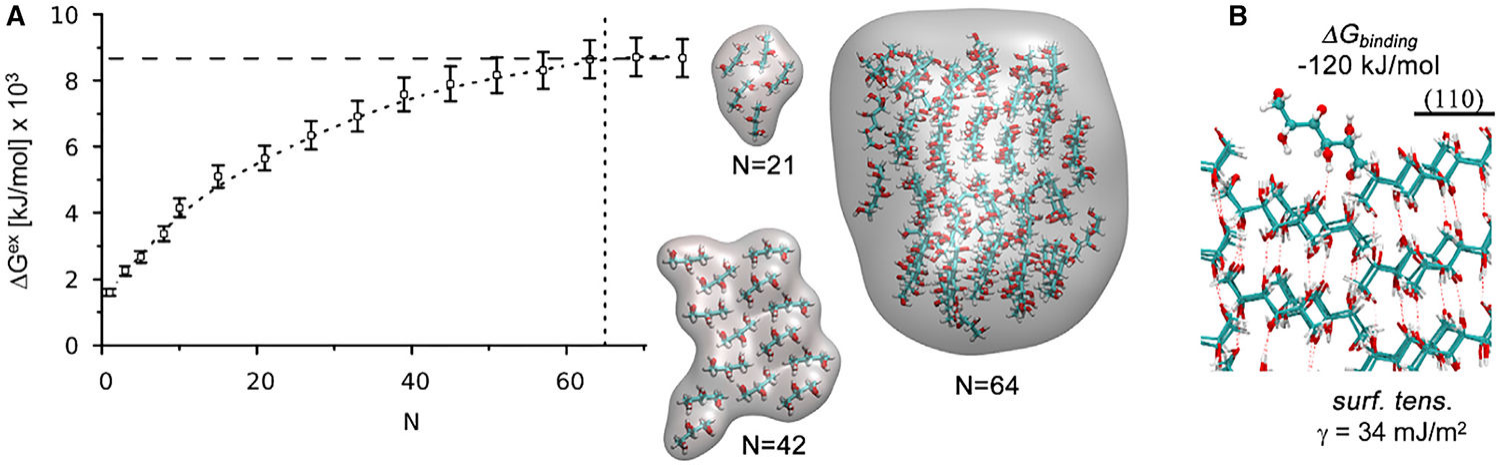
Thermodynamics of D-mannitol nucleation (A) Excess Gibbs energy *ΔG^ex^* of D-mannitol crystallites as a function of crystal size from restrained, equilibrium MD simulations analyzed with the 2PT Method. The results from our simulations (circles) are fitted to a natural log function (dashed line) to guide the eyes. The vertical dashed line at n = 64 is the predicted minimum crystallite size for spontaneous nucleation. Crystallites of up to 75 molecules were considered and found to have similar excess Gibbs energy to the 64 molecule case. The vertical dashed line is the Gibbs energy of a 2D periodic slab with the same number of D-mannitol molecules. Errors bars indicate the uncertainty in our calculated free energies (1 standard deviation) Insets: representative structures of various sized clusters. (B) Representative atomistic structure of a D-mannitol molecule at the (110) fast growing crystal face. The water molecules are not shown for clarity.

Classic nucleation theory^26^ posits that spontaneous crystal growth from solution will occur once a critical nucleus size forms by stochastic collisions of the (specific rotamer) molecules necessary for crystallization. The critical nucleus size is the point of vanishing chemical potential; i.e., *μ* = (∂*G_ex_*/∂*N*)*_T,P_* = 0. We verified that the nucleation kinetics of D-mannitol in the bulk solution is indeed classic^27^ by considering clusters with up to 70 molecules. While simulations capable of capturing the kinetics of nucleation on realistic timescales remains a grand challenge,^28^ we advance an approach here whereby we apply harmonic restraints to the center of mass of each molecule, in motifs seeded from the experimental crystal structure and calculate the excess Gibbs energy from equilibrium MD simulations combined with analysis using the two-phase thermodynamics method.^29^ Our simulations finds a critical nucleus size of N = 64 molecules (Figure 3). Indeed, unrestrained MD simulations showed that clusters with less than 64 molecules were unstable on the nano-second timescale of our MD simulations, while simulations with clusters of more than 64 molecules were stable (Figure S7 and the supplemental experimental procedures). We calculated a Gibbs binding energy of *ΔG_bind_* = −120 ± 5 kJ/mol at 277K for binding the key crystal forming rotamer to the (110) fast-growing crystal face, and we calculated a solid/liquid interfacial surface tension γ = 34 ± 4 mJ/m^2^. The calculated surface tension is within range of the experiment measurements^30^: 38 – 40 mJ/m^2^ at room temperature (Table S4).

Previous studies of freezing point depression by AFPs suggested that the IBS of AFPs interacts directly with the growing ice face to block growth^23,31^ or by a longer range effect whereby the vibrational dynamics of local water molecules are modified.^5^ However with micromolar concentrations of DAFP1, these mechanisms cannot explain the inhibition of 1M D-mannitol nucleation observed here. Thus to discover the mechanism, we first predicted^32^ the low-energy binding sites on DAFP1 for the CFR. We found two distinct binding sites with *ΔE*_*bind*_ = ~−75 kJ/mol (Table S5), each stabilized by at least 2 strong hydrogen bonds (HBs) to THR residues (Figure S8). The best binding pose was stabilized through HBs with the highly conserved T51, T53, T63, T65, and T77 residues on the well-defined IBS of DAFP1. Notably, the binding of other rotamers, specifically the BR, to DAFP1 is significantly less favorable than the CFR, due primarily to less favorable HBs.

### The catch switch and release mechanism for complete D-mannitol nucleation inhibition by DAFP1

**Figure 4.**
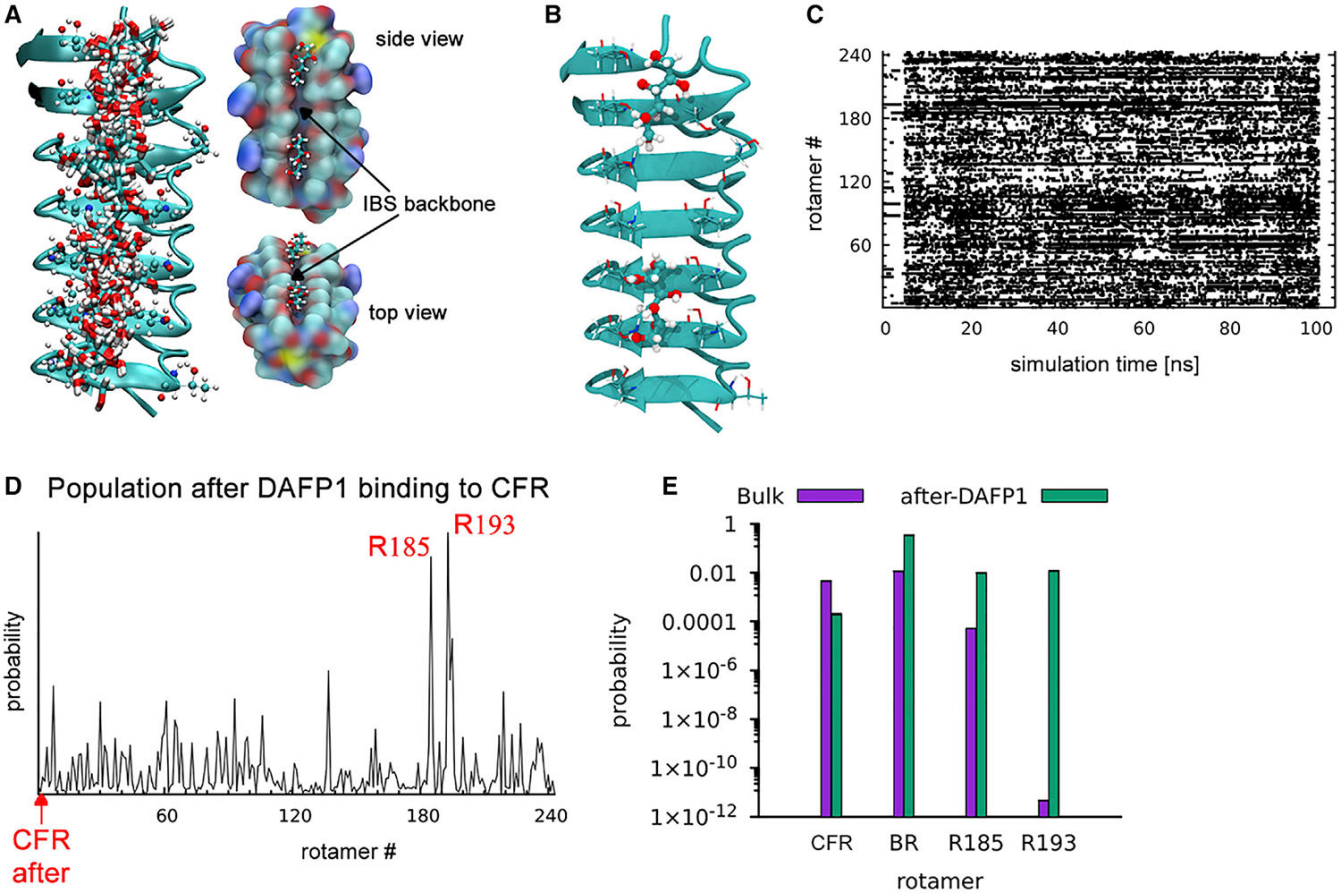
Mechanism of D-mannitol inhibition by DAFP1 (A) Molecular structure of D-mannitol in the IBS of DAFP1. Snapshots along our 100 ns MD simulations are superimposed, to demonstrate the mobility of D-mannitol along the backbone. Right insets: structure of two low energy binding sites. DAFP1 is represented by a surface electrostatic potential map. (B) Molecular structure of D-mannitol bound to both sites on DAFP1, stabilized by hydrogen bonds. (C) Rotamer map of a CFR molecule confined to the DAFP1 IBS. (D) Rotamer population analysis of (C), showing that the DAFP1 significantly reduces the CFR (rotamer #1 ) population. The most probable rotamer (#185 and #193) are also indicated. (E) Comparison of the predicated D-mannitol population at 277K in the bulk solution (purple bars) and after application of 1mM DAFP1 (green bars). Data for the crystal forming rotamer (CFR), best bulk rotamer (BR), and rotamers #185 and #193 are presented along with the calculated error bars (representing 1 standard deviation). Note the log scale on the y axis for the populations, where the CFR population is reduced by a factor of 2,500 in the presence of DAFP1 however the BR population is relatively unaffected.

Extensive MD simulations, with the CFR D-mannitol confined to the IBS of DAFP1 in explicit solvent, revealed strong binding to the IBS (Figure 4A). Here, the D-mannitol molecule walks randomly along the IBS backbone during MD due to thermal fluctuations, after an initial 5 ns delay during which the bound D-mannitol adjusts to the solvent environment. Notably, the *Cis* configurations for some of the D-mannitol O-C-C-O torsional angles are stabilized (see the spikes at 0° for all 5 dihedrals in Figure S9 and the supplemental experimental procedures), due to enhanced H-bonding to DAFP1 (Figure 4B), dramatically reducing barriers for torsional transitions (Figure S10 and the supplemental experimental procedures). In fact, we find that D-mannitol on the DAFP1 surface switches conformations at a rate of 10 THz (Figure 4B). Although *Cis* configurations lead to high internal energy for the isolated D-mannitol, they increase HBs with adjacent hydrophilic groups on the DAFP1 IBS. Thus, the nanoscale architecture of the IBS of DAFP1, with ladder-like placement of THR and SER groups, facilitates new HBs to the key CFR, causing the rapid conformational switching that depresses the population of the D-mannitol CFR. Notably, we find that the structure of the IBS is generally well preserved, even while binding the D-mannitol (Figure S11 and the supplemental experimental procedures), consistent with X-ray crystallography studies that show that all the side chains of the THR residues have identical conformations. The net result is rapid switching to new conformations, with two distinct, otherwise high-energy, conformations particularly favored on the DAFP1 surface: #193: N-N-N-P-N (1.2%) and #185: N-P-T-N-P (1.0%) (Figure 4C). This nanoscale architecture, that facilitates the rapid conformer switching, is destroyed in the denatured DAFP1 and is not present in either AFGP4-5 or AFGP8, which explains why the AFGPs did not have the same nucleation inhibitory ability.

**Figure 5.**
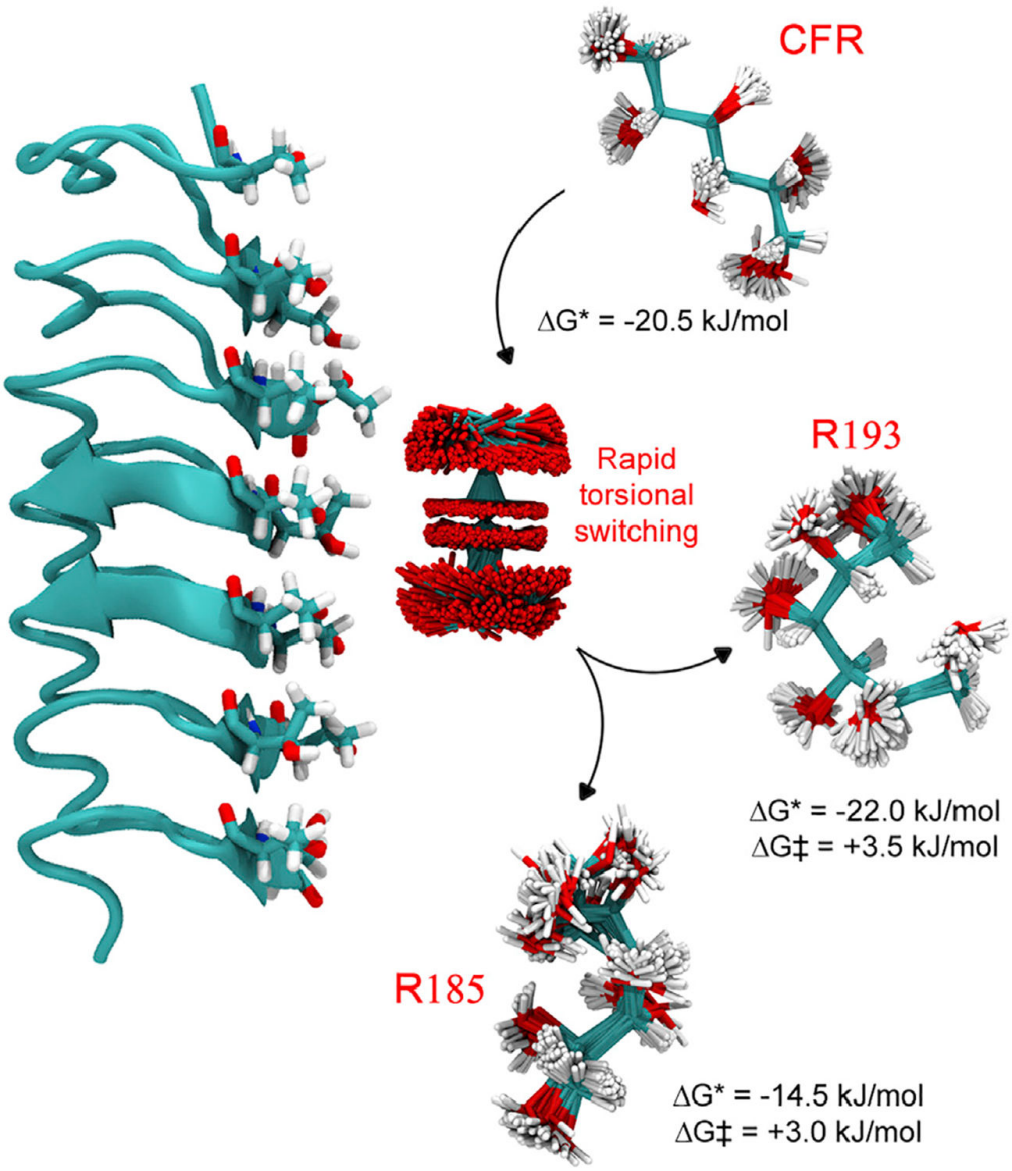
Molecular mechanism of D-mannitol crystallization by DAFP1 Schematic of catch switch and release (CSaR) mechanism. The molecular structures are shown. In the case of D-mannitol (ball and stick representation), the fluctuations in the structure are presented. The binding Gibbs energy (ΔG*) and activation barrier (ΔG^#^) are reported.

We further quantified the binding thermodynamics of the various rotamers to DAFP1 by means of accelerated MD simulations, which showed a Gibbs energy of binding *ΔG_bind_* = −20.5 kJ/mol for the CFR. In contrast, the binding free energy of two of the most likely conformers on the DAFP1 surface, #185 and #193, have unfavorable binding energies of *ΔG*_*bind*_ = +14.5 and +22.0 kJ/mol, respectively, and relatively modest desorption barriers: *ΔG^#^* = +3.0 and +3.5 kJ/mol respectively (Figure S12 and the supplemental experimental procedures). Unrestrained MD simulations support our free energy analysis: we find 3 desorption events over 100 ns of MD simulation, all of which were initiated by either rotamer #193 or #185. Overall, the computational results are summarized as the catch switch and release (CSaR) mechanism (Figure 5): DAFP1 preferentially binds the CFR molecule in the bulk solution, randomizes the torsions, and populates unfavorable configurations that desorb stochastically from the surface. Due to the large torsional rotational barriers in the bulk, these unfavorable configurations have relatively long lifetimes. The net result is that the total population of CFR molecules in the bulk is reduced by over three orders of magnitude, from 0.45% to ~2.0 × 10^−4%^ (Table S6 and the supplemental experimental procedures). Thus, the CSaR mechanism explains that micromolar DAFP1 is sufficient to inhibit D-mannitol nucleation. Assuming a linear relationship between the concentration and nucleation rate,^26^ we predict that this reduction in the CFR population would extend the timescale for forming the critical nucleus from 14 days to nearly ~75 years.

In summary, we demonstrated experimentally that the stability of a highly supersaturated D-mannitol solution can be extended very dramatically by a hyperactive insect AFP, DAFP1, at a micromolar level. Extensive computer simulations suggest a new molecular mechanism that elucidates the origin for this unprecedented effect. These results advance a previously unreported and underappreciated role of AFPs in stabilizing highly supersaturated osmolyte solutions, suggesting new applications for these natural polymers. For example, isoforms of DAFP in the hemolymph of *D. canadensis,*^33^ which are expected to have similar nanoscale architecture as that of DAFP1, may be other potential effective nucleation inhibitors for D-mannitol or analogs. It should also be noted that DAFP1 is used as an external additive here and may need to be removed from the D-mannitol solution through ultrafiltration until the safety of its inclusion is fully evaluated. By revealing the inventiveness of nature, these findings also suggest new design principles in controlling the crystal nucleation processes (especially those of small organic compounds), where conformers play a key role.

## EXPERIMENTAL PROCEDURES

### Resource availability

#### Lead contact

Further information and requests for resources should be directed to and will be fulfilled by the lead contact, Xin Wen (xwen3@calstatela.edu).

#### Materials availability

This study did not generate new unique materials.

#### Data and code availability

The data that support the findings of this study will be made available over the web. In-house programs that implement the two-phase thermodynamics method are available from the corresponding authors upon request. Modification to the LAMMPS simulation engine used in this study will be submitted to the original developers for inclusion in the official release.

### Materials

Chemicals were purchased from Sigma-Aldrich (St. Louis, MO) at ACS grade or better, except β-D-galactopyranosyl-(1- >3)-2-acetamido-2-deoxy-α-D-galactopyranose (GalNAc, analytical grade) was ordered from Santa Cruz Biotechnology (Santa Cruz, CA) and were used without additional purifications. In particular, solvents and chemicals for the HPLC experiments were purchased at HPLC grade from Sigma-Aldrich (St. Louis, MO). Aqueous solutions were prepared using Milli-Q water produced from a Synergy water system (Millipore) with a minimum resistivity of 18 MΩ•cm. Unless otherwise indicated, all of the samples including the proteins and peptide samples were filtered through 0.1 mm filters before use. Sample vials (8 mL, National Scientific) were purchased for crystallization. All glassware and stir bars used for crystallization were extensively washed. Briefly, they were first cleaned in a KOH/2-propanol bath. After rinsed with distilled water, they were soaked in 1 M HCl for 24 h. After rinsed with distilled water, they were finally cleaned using RBS 35 (Thermo Fisher), a surface-active detergent. After rinsed with distilled water and then with deionized water thoroughly, the washed glassware and stir bars were air-dried at room temperature before use.

### Antifreeze proteins preparation

DAFP1, an AFP from *Dendroides canadensis,* was expressed and purified as described previously.^12^ Briefly, the protein was expressed as a fusion protein in *Escherichia coli.* The cells were harvested by centrifugation at 4°C. After the cells were disrupted, the crude protein was purified using immobilized metal ion affinity chromatography (IMAC) (Ni-NTA agarose, QIAGEN). The tags of the AFP were cleaved off enzymatically and the tag-free protein was further purified by using IMAC and ion exchange chromatography. The purified DAFP1 was characterized by the matrix-assisted laser desorption ionization time-of-flight (MALDI-TOF) mass spectrometry, circular dichroism spectrometry, and differential scanning calorimetry as described previously,^34^ and the identity of DAFP1 was confirmed. The purity of DAFP1 was assessed by high-performance liquid chromatography (HPLC) with a purity higher than 95%. The concentration of stock DAFP1 solution or stock denatured DAFP1 was determined using a Cary 100 Bio UV-Vis spectroscopy (Varian) and the extinction coefficient of 5.47 × 10^3^ M^−1^cm^−1^ at 280 nm was used.^34^ The denatured DAFP1 with completely reducing its disulfide bonds was prepared following a known methods.^10^ Briefly, 1.0 mM purified DAFP1 was incubated in 0.10 M sodium citrate, pH 3.00, and 15.0 mM tris(2-carboxyethyl)phosphine hydrochloride (TCEP) for 30 min at 60 °C. The denatured DAFP1 was purified using ÄKTA Purifier 10 (GE Healthcare) with a Sephacryl S-100 gel filtration column (GE Healthcare). Antifreeze glycoproteins, AFGP4–5 and AFGP8 from the Antarctic notothenioid *Trematomus borchgrevinki,*^9^ were gifts from Professor Yin Yeh (UC Davis). The AFGPs were received as lyophilized powders and were used without further purification. All the weight measurements for AFGPs were carried out with an Ohaus Voyager Pro analytical and precision balance (Parsippany, NJ).

### Crystal growth procedure

Slow evaporation of a D-mannitol aqueous solution can yield β-form D-mannitol crystals, but it produces large amounts of microcrystals.^20^ In order to yield pure β-form D-mannitol crystals without microcrystals, we tested many crystallization conditions and found that lowering the growth temperature can eliminate the microcrystals and yield large pure β form D-mannitol crystals. The test was repeated seven times and the crystallization of D-mannitol alone was reproducible with respect to the sizes, shapes, and final weights of the final crystals. The developed crystallization conditions were then used in the following experiments for D-mannitol alone and in the presence of AFPs and controls, respectively.

Briefly, on day 1,20 μL of either water or 2-acetamido-2-deoxy-3-O-(β-D-galactopyranosyl)-D-galactose, denatured DAFP1, DAFP1, AFGP4-5, and AFGP8 solutions at specific concentrations, respectively, were added into each sample vial containing 2.00 mL of supersaturated D-mannitol aqueous solution. The final D-mannitol concentration was 1.00 M in each vial. The final molar ratios of the controls (i.e., GalNAc, denatured DAFP1) to D-mannitol were 1.0 × 10^−2^ in the vials. The final molar ratios of AFPs to D-mannitol in the vials were either 1.0 × 10^−2^ or 2.8 × 10^−6^ for DAFP1; and either 1.0 × 10^−2^ or 2.8 × 10^−5^ for AFGP4-5 or for AFGP8. The vials were gently swirled after the additions. The sample vials were then tightly closed using caps and stored at 4 °C. Three observations were recorded for each vial every day since day 1 until no apparent growth of crystals was observed. The intervals between two consecutive observations were about 8 h, but not less than 6 h. All the experiments were repeated seven times. Sample results were listed in Table S1.

The crystallization in the vials except those in the presence of DAFP1 were stopped after 200 days at 4°C by separating the mother liquor from the formed crystals. The vials were then warmed up to room temperature and photos of the vials were taken. The crystals were dried at room temperature for optical microscopy and other characterizations. Microscopic observations were performed with a Nikon SMZ-1000 polarizing microscope equipped with a DS-Fi2 color camera after the crystallization were completed. The weights of the achieved crystals were analyzed for five experiments in comparison to the total amounts of D-mannitol added into the vials, and the average values were listed in Table S1.

### NMR spectroscopy

Solid-state ^13^C cross-polarized magic angle spinning (^13^C CP/MAS) NMR spectra were recorded at 298 K at 75.47 MHz (^13^C) on a Bruker spectrometer using a 4 mm broadband MAS probe with proton broadband decoupler. Approximately 120 mg of solids were gently ground using mortar and pestle and packed in a 4 mm wide ZrO_2_ rotor with a Kel-F cap. Spinning frequency of 10 kHz, CP contact time of 1.5 ms, and a 60 s delay were utilized.

### Equilibrium MD simulations

Molecular dynamics (MD) simulations were performed using LAMMPS.^35^ The D-mannitol molecule was described with the CHARMM carbohydrates forcefield,^36^ with parameters obtained from the Ligand reader and modeler input generator^37^ on the CHARMM-GUI web portal.^38^ The water molecules were described using the TIP4P-ice water model.^39^ The DAFP1 starting structure was obtained from our previous work^34^ and described here in fully atomistic detail using the CHARMM36m^40^ plus CMAP^41^ forcefields.

In each simulation, we employed a 1.2 nm distance cutoff for the van der Waals interaction, where the energy and forces after 1.0 nm go smoothly to zero using a cubic spline switching function. The real space cutoff for the electrostatics was also 1.2 nm, and the long-range electrostatic interactions were obtained from the particle-particle particle-mesh method,^42^ with a force tolerance of 10^−6^.

In our equilibration MD procedure, we first performed an initial energy minimization at 0 K and then slowly heated from 0 K to 277K at constant volume over 0.5 ns using a Langevin thermostat, with a damping parameter of 100 ps. The system was then subjected to 5 cycles of quench-annealing dynamics, with a 500 kcal/mol/Å^2^ spring applied to the protein/ligand complex to keep it from moving. After annealing, the restraints were removed and the system was equilibrated using the constant temperature (277 K), constant pressure (1 bar) (NPT) ensemble for 1 ns. Finally, we simulated the system in the NVT ensemble for at least 20 ns, saving snapshots of the system (atomic positions and coordinates) every 1ps.

### Accelerated meta-dynamics simulations

We explored the rugged free energy landscape for three systems using well-tempered^43^, multiple walker^44^ metadynamics^24,45,46^: (1) the β, δ torsional rotation 2D PES of D-mannitol in the bulk solvent, (2) the 2D PES of D-mannitol confined to the DAFP1 surface, and (3) the binding thermodynamics various D-mannitol rotamers to the DAFP1 binding site 1. In all cases, we use 10 walkers, each initiated from various points along the equilibrium production MD trajectory. In cases 2 and 3, we further accelerated convergence by restraining the motions of the D-mannitol molecule using the funneling approach.^47^ In case 3, we separately considered the various rotamers by constraining the D-mannitol internal structure using rigid body dynamics.^48^

In each simulation, MW-wt-MetaD biases were constructed as follows: Gaussian functions were deposited every 0.5 ps with an initial height of 277/T x 1.0 kcal/ mol. The bias factor [g = (T + ΔT) / T] was set to 5. The widths were 0.01 and 0.1 for the torsion rotation barriers and the center of mass distance between the DAFP1 and D-mannitol, respectively. We monitored convergence by calculating the free energy profiles every 1 ns and found that ~30 ns was reasonable in most cases. All simulations were performed using LAMMPS and Plumed 2.5.^49-51^

## Supplementary Material

1

## Highlights

Spontaneous crystallization of a polyol is halted by a natural biopolymer

The insect antifreeze protein (DAFP1) completely inhibits D-mannitol nucleation

DAFP1 recognizes and reduces D-mannitol crystal forming rotamer (CFR) in solution

The reduction in the CFR population in solution extends the nucleation time
