# Supplementary material for: Complete inhibition of a polyol nucleation by a micromolar biopolymer additive
Source: Cell Rep Phys Sci. Author manuscript; Available in PMC 2022 Mar 8. (PMC8903182; doi:10.1016/j.xcrp.2021.100723)
Supplement: 1 [file NIHMS1781668-supplement-1.pdf]

Cell Reports Physical Science, Volume *3*

Supplemental information

Complete inhibition of a polyol nucleation by a micromolar biopolymer additive

Xin Wen, Sen Wang, Robert Ramji, Luke O. Butler, Yelena Bagdagulyan, Audrey Kishishita, James A. Golen, Arnold L. Rheingold, Soo-Kyung Kim, William A. Goddard III, and Tod A. Pascal

Supplementary Experimental Procedures

Expanded experimental and computational method.

Experimental Materials and Methods.

a. Fourier Transform Infrared (FTIR) Spectroscopy

The FTIR attenuated total reflectance (ATR) spectra of the crystalline mannitol were collected on a Nicolet iS5 FT-IR spectrometer (Thermo Fischer, Waltham, MA) equipped with an iD5 ATR accessory. The IR frequencies were recorded in cm^{-1} and the spectra were measured in a spectral range from 4,000 to 200 cm^{-1}.

b. Crystal growth procedure

Seeding dissolution experiments were performed to check whether the additives affect the solubility of the D-mannitol solution. The same tiny amount of D-mannitol seed solids, 11.00 mg, were added into 4 °C preequilibrated 1000 µL 1.00 M D-mannitol supersaturated solutions in the absence and presence of additives of DAFP1 at 2.8 $\times 10^{-6}$ M, AFGP4-5 at 28 $\times 10^{-5}$ M, and AFGP8 at 28 $\times 10^{-5}$ M, respectively. Here, the D-mannitol chemical for D-mannitol solution preparation was used as seeds. Except the D-mannitol solution in the presence of DAFP1 (used the ones that made about three years ago, but without nucleation), all the other samples were prepared freshly. After 24 hours, the solutions were centrifuged at 4 °C and the supernatants were removed. The remained solids were washed three times using 1.00 M D-mannitol supersaturated solution at 4 °C. The washes were removed by centrifugation. After the solids were dried (lyophilized) till no more mass changes, the weights of the solids were recorded. The solids were then dissolved in 450 µL water (HPLC grade) and 50 µL D_2O. The solutions were then subjected to the analyses by ^1H and ^{13}C solution NMR spectroscopy on a Bruker 400 NMR spectrometry and high-performance liquid chromatography (HPLC) on a Waters HPLC system consisting of a Waters 1525 binary HPLC pump, a Xbridge BEH200, 3.5 µm SEC HPLC column, 7.8 × 150 mm (Waters, Milford, MA) and a Waters 2998 photodiode array detector at room temperature. The results of solution NMR confirmed that the solids are D-mannitol and no protein residues were detected by HPLC (data are not shown). The experiments were repeated three times.

c. NMR Spectroscopy

Bruker 400 NMR spectrometry was used to acquire ^1H NMR (10% D_2O, 400 MHz NMR) and ^{13}C NMR (10% D_2O, 100.6 MHz NMR) at 293 K. The ^1H and ^{13}C NMR spectra of the D-mannitol solutions in the presence of DAFP1 (stored for almost 3 years at 4 °C) match the standard spectra of D-mannitol and are identical to those of freshly made D-mannitol solution in the absence of additives confirming the integrity of D-mannitol in the solution.

d. Single Crystal X-ray Diffraction

The D-mannitol crystals obtained in the absence and presence of additives sent to X-ray crystallography laboratory at UCSD, where they were checked and confirmed to be β-form D-mannitol crystals. The D-mannitol crystals achieved in the presence of denatured DAFP1 was analyzed. Colorless crystal of D-mannitol was mounted on a Cryoloop with Paratone-N oil and data was collected at 100K with a Bruker APEX II CCD using Cu K alpha radiation. All non-hydrogen atoms were refined anisotropically by full matrix least squares on F^2. Hydrogen atoms (H1 – H6) were found from Fourier difference map and were refined with O-H distance of 0.86 (0.01) Å and 1.50 U_{eq} of parent O atoms. All other hydrogen atoms were placed in calculated positions with appropriate riding parameters. Additional information on data collection parameters is given in **Table S2**. The crystallographic data of D-mannitol crystals grown in the presence of denatured DAFP1 was deposited in the Cambridge Database (CCDC) and the CCDC deposit number is 2015391. The data are in good accordance with those of β-form D-mannitol published previously[1] with improved resolution and were used for structural analysis of the fastest growth faces and the crystal forming rotamer (CFR) of D-mannitol.

Theoretical and Computational Methods

a. The DarwinDock Method for Predicting Ligand-Protein Structures

To predict the binding site of D-Mannitol to DAFP1 in vacuum, we use the DarwinDock complete sampling method[2]. The procedure as follows: we start with the structure of the D-mannitol crystal forming rotamer (CFR) and the best energy rotamer the bulk solution (BR). We used DOCK4.0[3] to generate ~50,000 poses (but without energy calculations) sufficient to span the putative binding regions of the alanized protein in which the 6 hydrophobic residues are replaced with alanine. To ensure complete sampling, these poses are generated in increments of 5,000 and clustered into Voronoi families based on RMSD until <2% new families are generated. The family heads are then energy scored using the Dreiding force field[4] selecting with the top 10% by energy. Then we calculated the binding energy for all members of these top 10% families and selected the lowest 100 poses for further optimization. At this point we dealanized using SCREAM (Side Chain Rotamer Excitation Analysis Method[5]) which is optimized for optimum interactions between nearby side chains. This allows the side chains to adjust individually to each pose[2]. We then minimize the whole complex for 100 steps to an RMS force of 0.5 kcal/mol/Å. The energetically most favorable docked structures were selected for further MD simulations. We found two isoenergetic binding sites for D-mannitol on DAPF1 (**Figure S7**). Energy decomposition analysis of the best poses for each case is presented in **Table S5**.

b. Rotamer energies from Quantum Chemistry calculations

Starting with the crystal Nucleus Formation rotamer (NFR), we generated 242 additional rotamers of D-mannitol by rotating about each of the 5 central C-C bonds sequentially, using the Confab utility (no energy or RMS cutoffs) in Openbabel 3.0.1.[6] The positions of the H and OH group on each of the rotamers were then optimized in the Q-Chem 5.1 quantum chemistry package[7] at the MP2/cc-pVTZ[8,9] level of theory. Practically, this amounted to placing constraints on each of the 5 torsion angles during optimization. The energy of each rotamer is presented in the **Table S3**. Here the NFR is labelled #1, the best bulk rotamer, BR, is labelled #19.

c. Equilibrium MD Simulations

Molecular Dynamics (MD) simulations were performed using LAMMPS[10]. The D-mannitol molecule was described with the CHARMM carbohydrates forcefield,[11] with parameters obtained from the Ligand reader and modeler input generator[12] on the CHARMM-GUI web portal.[13] We first tested the ability of the forcefield to match relative rotamer energies from QM, by performing single point calculations in vacuum. Overall, we found good agreement between the forcefield and QM. The water molecules were described using the TIP4P-ice water model,[14] optimized to reproduce the thermodynamics of ice and amorphous low temperature water. Notably, the melting temperature of this water model is 270K, in very good agreement with the experimental value of 273.15K. Our approach mirrors a previous study examining AFP binding to ice crystals.[15] We extended the LAMMPS code to allow for the TIP4P-ice water model in the CHARMM forcefield. These improvements can be provided upon request and will be submitted to the LAMMPS developers to be included in the standard release. We further tested the accuracy of our forcefield by comparing various thermodynamic properties of the respective crystals to experiment at 277K. Overall, we find good agreement (**Table S4**).

The DAFP1 starting structure was obtained from our previous work[16] and described here in fully atomistic detail using the CHARMM36m[17] plus CMAP[18] forcefields. The atom and residue names and partial atomic charges were obtained from the PDB Manipulator utility[19] on the CHARMM-GUI web portal. The disulfide bonds between adjacent cysteine residues: 76↔82, 64↔70, 52↔58, 40↔46, 28↔34, 15↔21, 8↔18 and 2↔11, were all maintained in the native structures. We verified our native DAFP1 model by performing equilibrium MD simulations in bulk solvent and 0.1M NaCl salt, which resulted in a coordinate root mean square deviation of 1.2Å from the starting structure, and an average of 0.5Å from the equilibrated MD structure. The NaCl salt atoms were described with the optimized parameters of Roux et al.[20]

In each simulation, we employed a 1.2nm distance cutoff for the van der Waals interaction, where the energy and forces after 1.0nm go smoothly to zero using a cubic spline switching function. The real space cutoff for the electrostatics was also 1.2nm, and the long range electrostatic interactions obtained from the particle-particle particle-mesh method,[21] with a force tolerance of 10^{-6}.

For simulations of the isolated D-mannitol rotamers, we immerse each rotamer in a preequilibrated box of 700 TIP4P-ice water molecules, removed all water molecules within 3Å of the D-mannitol surface, and adjusting the final number of water molecules to 648. The initial cell lengths were 27.4 x 26.0 x 29.1 $Å^3$. For simulations of the DAFP1, we immersed the initial system in a pre-equilibrated box of 9650 TIP4P-ice water molecules (initial cell dimension 62.5 x 46.0 x 60.5 $Å^3$). Water molecules within 4Å of the protein/ligand surface were removed and we first neutralized the system by adding 1 Na+ ion. In this procedure, we calculated the electrostatic potential of the protein/ligand complex using the APBS software package[22], and replaced the water molecule nearest to the region of most negative potential with a Na^+ ion. This was followed by a similar procedure to place 0.01 M NaCl (10 atoms each), after which we adjusted the total water molecules to be 5555.

Our equilibration MD procedure is based on our standard approach[23-35]. We first performed an initial energy minimization at 0 K (energy and force tolerances of 10^{-4}) using the conjugate gradient minimization scheme to obtain the ground-state structure. Then, the system was slowly heated from 0 K to 277K at constant volume over 0.5 ns using a Langevin thermostat, with a damping parameter of 100 ps. The system was then subjected to 5 cycles of quench-annealing dynamics, with a 500 kcal/mol/$Å^2$ spring applied to the protein/ligand complex to keep it from moving. Here, the temperature was slowly cycled between 277 K and 894 K over 1 ns using a Nose-Hoover thermostat (temperature relaxation constant of 100 fs), in order to eliminate the persistence of any meta-stable states. The equations of motion were integrated by means of the velocity verlet algorithm using a 1 fs timestep. After annealing, the restraints were removed and the system was equilibrated using the constant temperature (277 K), constant pressure (1bar) (NPT) ensemble for 1 ns. We resolved stresses in the system anisotropically using the Andersen barostat (pressure relaxation constant of 1 ps). We used the Shinoda et al.[36] equations of motion which combine the hydrostatic equations of Martyna et al.[37] with the strain energy proposed by Parrinello and Rahman[38]. The time integration schemes closely follow the time-reversible measure-preserving Verlet integrators derived by Tuckerman et al.[39]. During the last 0.5 ns of the 1 ns NPT simulation, we calculated the average cell lengths and linearly adjusted the final NPT simulation cell to that average over a further 0.1 ns of dynamics. This was followed by a 1ns of NVT dynamics for equilibration. Finally, we simulated the system in the NVT ensemble for at least 20ns, saving snapshots of the system (atomic positions and coordinates) every 1ps for visualization using VMD[40] and analysis with various in-house Python scripts.

d. Gibbs energy from the Two-Phase Thermodynamics Method

Starting with snapshots of each system every 1ns from the final NVT production dynamics, we ran an additional 50ps NVT simulation, this time saving the velocities and coordinates every 5fs (10,000 frames in the corresponding trajectory). Each trajectory was then analyzed by an in-house code[41] that implements the 2PT method.[42-46] When performing the 2PT analysis, we separately considered the thermodynamics of the various groups (water, ions, D-mannitol and DAFP1) separately, where appropriate. The final values for the total entropy, quantum corrected enthalpy and Gibbs energy were taken as statistical averages over the independent, 1ns separated trajectories. We note that as implemented, in the canonical ensemble the fundamental thermodynamic observable is the Helmholtz energy, which we report as the Gibbs energy since the PdV correction term is negligible.

We use the 2PT approach to calculate the excess Gibbs energy ΔG^{ex} of various sized D-mannitol clusters in solution (**Figure S4**). In these simulations, we constrained the center of mass of each D-mannitol molecule by zeroing the x,y,z components of the solute's momentum vector each timestep. This prevented the dissolution of clusters below the critical nucleus size. We then calculated $\Delta G^{ex} = G(system) - G(bulk - water) - G(dman - xtal)$, where G(system) is the total Gibbs energy of the solvated cluster, G(bulk-water) is the energy of bulk water bulk with the same number (648) of TIP4P-ice water molecules, and G(dman-xtal) is the energy of N D-mannitol molecules in the bulk crystal from a separate independent run.

We also used the 2PT energies to calculate the solid/liquid surface tension γ of the (110) plane for the D-mannitol fast growing crystal phase. Here we follow our previous approach[25,27]:

$$\gamma = \frac{\Delta G^{ex}}{S.A.} = 166.03 * \frac{G(system) - G(bulk)}{S.A.}$$

e. Accelerated Meta-Dynamics Simulations

The potential energy surface of D-mannitol is rugged, containing various metastable states with deep minima which could make exploring all conformational states using traditional MD simulation prohibitive. One way of overcoming this is through enhanced sampling, and in particular, the recently developed Metadynamics approaches.[47-49] We employed two recent advances in order to accelerate convergence: **a**) we used the well-tempered formulation,[50] where the bias deposition rate decreases over time by rescaling the heights of the deposited Gaussian functions; and **b**) we used the multiple walker scheme[51] with well-tempered simulations (MW-wt-MetaD).

We explored three systems with this approach: **1**) the β, δ torsional rotation 2D PES of D-mannitol in the bulk solvent (**Figure S3**), **2**) the 2D PES of D-mannitol confined to the DAFP1 surface (**Figure** S9), and **3**) the binding thermodynamics various D-mannitol rotamers to the DAFP1 binding site 1 (**Figure S10**), using the center of mass of the D-mannitol and residues comprising the binding site 1 of DAFP1. In all cases, we use 10 walkers, each initiated from various points along the equilibrium production MD trajectory. In cases 2 and 3, we further accelerated convergence by restraining the motions of the D-mannitol molecule using the funneling approach[52]: constraining the center of mass to be within 0.5nm of the DAFP1 surface in case 2, and to within a 0.5 nm^2 region around site 1 in case 3 (**Figure S10b**). In case 3, we separately considered the various rotamers by constraining the D-mannitol internal structure using rigid body dynamics.[53]

In each simulation, MW-wt-MetaD biases were constructed as follows: Gaussian functions were deposited every 0.5 ps with an initial height of 277/T x 1.0 kcal/mol. The bias factor [$\gamma = (T + \Delta T) / T$] was set to 5. The widths were 0.01 and 0.1 for the torsion rotation barriers and the center of mass distance between the DAFP1 and D-mannitol respectively. We monitored convergence by calculating the free energy profiles every 1ns (**Figure S10e**) and found that ~ 30ns was reasonable in most cases. All simulations were performed using LAMMPS and Plumed 2.5.[54-56]

f. Population Analysis

For a closed system at constant temperature (i.e. constant number of particles N, volume V and temperate T), the total number of accessible (micro-) states is given by the partition function Q:

$$Q = \sum_{v=1} \exp(-\beta E_v) \quad (3)$$

where $\beta = 1/T$ and E_v is the energy of the state. When considering the D-mannitol rotamers, equation 3 becomes:

$$Q = \sum_{v=1}^{243} \exp(-\beta E_v) \quad (4)$$

and the probability of observing a particular rotamer i is

$$P_i = \exp(-\beta E_i) \Big/ Q = \exp(-\beta E_i) \Big/ \sum_{v=1}^{243} \exp(-\beta E_v) \quad (5)$$

In this work, the total internal energy of the isolated D-mannitol rotamer is obtained from either QM calculations (**Section h**) or from the forcefield (**Section i**). In the case of the forcefield, the energies are obtained in the vacuum a 0K (to compare directly to QM) and in the bulk solution at 277K (to predict the population distribution in experiments).

Supplementary Figures

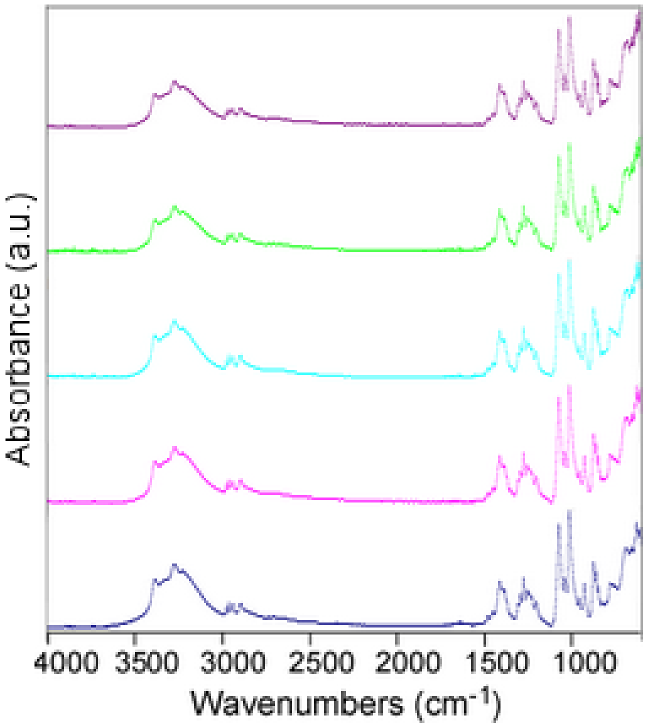

Figure S1. Representative ATR-FTIR spectra of finally achieved D-mannitol crystals that were achieved from the solutions of D-mannitol in the presence of GalNAc (purple), in the absence of additives (green), in the presence of denatured DAFP1 (cyan), AFGP4-5 (pink), and AFGP8 (blue), respectively. The spectra of the achieved D-mannitol crystals are in good agreement with published data for β-form D-mannitol,[57] indicating that all crystallites are pure β-form D-mannitol.

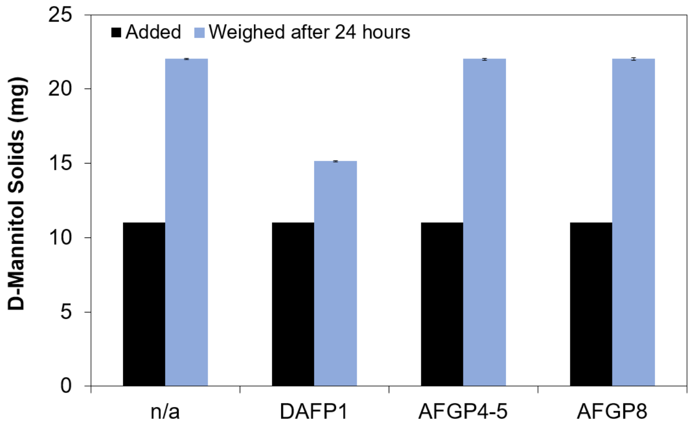

Figure S2. Seeding experiments with the same excess amount of D-mannitol added into D-mannitol supersaturated solutions in the absence and presence of additives. The solutions are 1.00 M D-mannitol (n/a), 1.00 M D-mannitol in the presence of 2.8 $\times 10^{-6}$ M DAFP1 (DAFP1), 2.8 $\times 10^{-5}$ M AFGP4-5 (AFGP4-5), and 2.8 $\times 10^{-5}$ M AFGP8 (AFGP8), respectively. Black bars represent the amount of seed D-mannitol added into each solution. Light blue bars represent the number of D-mannitol solids harvested after 24 hours. The experiments were performed at 4 °C. Error bars are 3 standard deviations.

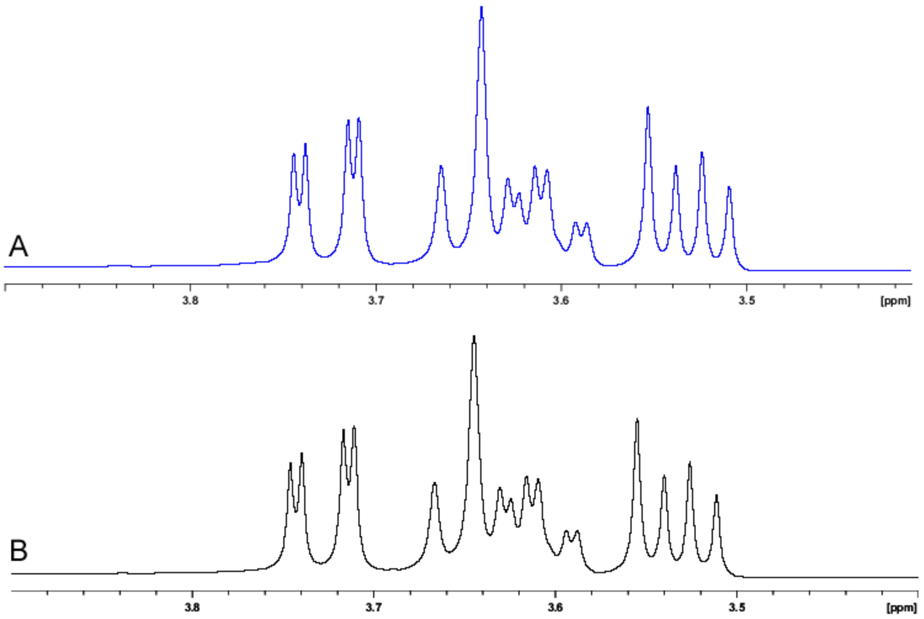

Figure S3. Representative ^{1}H NMR spectra of D-mannitol solution (black) and D-mannitol solution in the presence of 2.8 ×10^{-6} μM DAFP1 (blue). The sample of D-mannitol solution was freshly prepared, while the sample of D-mannitol solution in the presence of 2.8 ×10^{-6} μM DAFP1 was the one that made about three years ago, but without nucleation.

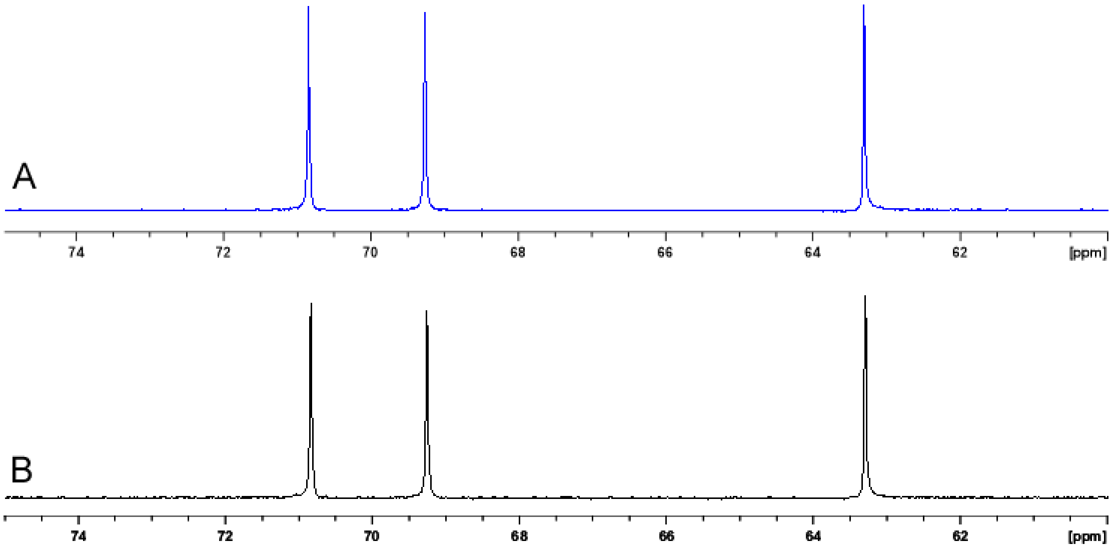

Figure S4. Representative ^{13}C NMR spectra of D-mannitol solution (black) and D-mannitol solution in the presence of 2.8 ×10^{-6} μM DAFP1 (blue). The sample of D-mannitol solution was freshly prepared, while the sample of D-mannitol solution in the presence of 2.8 ×10^{-6} μM DAFP1 was the one that made about three years ago, but without nucleation.

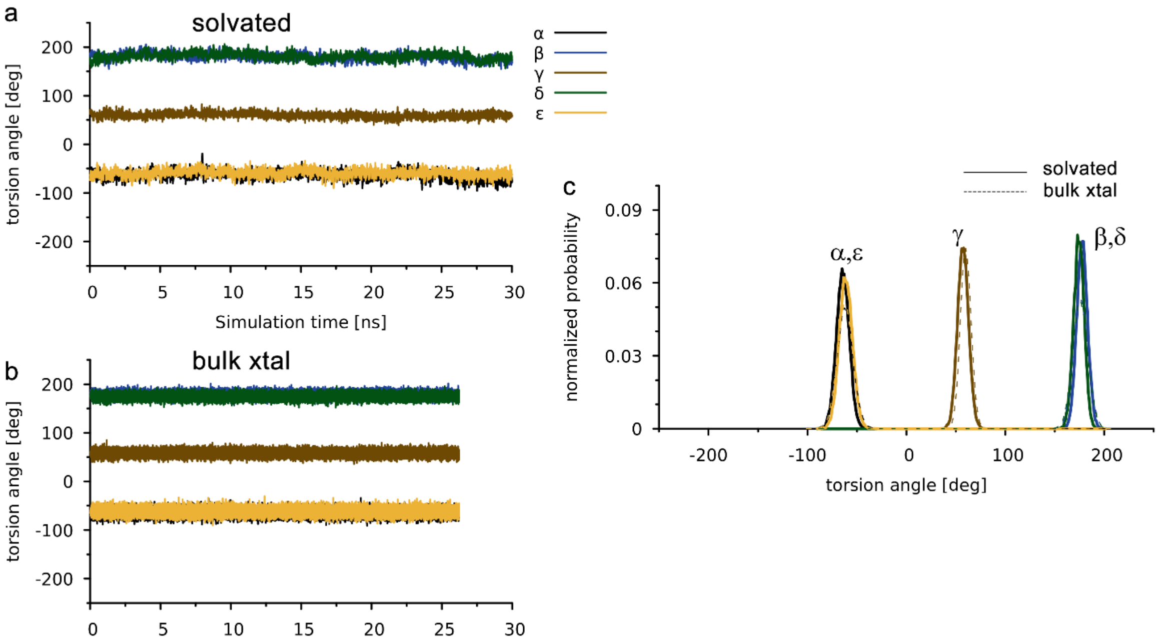

Figure S5. Mechanical stability of D-mannitol molecules. a, Time progressions of each of the 5 torsional angles (as labelled) of the crystallization rotamer of D-mannitol from a 30ns bulk solvent equilibrium MD simulation. **b,** Time progression of the torsional angles in the bulk crystal from a 25 ns equilibrium MD simulation. **c,** Distribution of the torsional angles for the solvated (solid lines) and crystal (dashed lines) MD simulations. The color scheme is the same as in a.

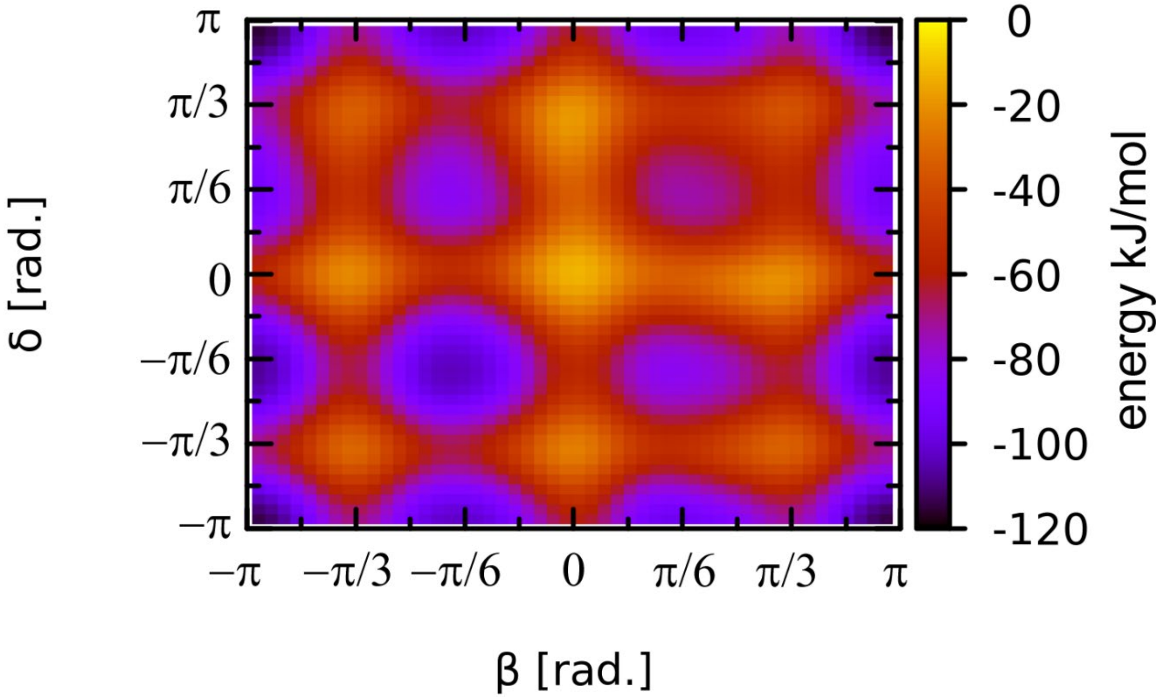

Figure S6. D-Mannitol torsional rotation barriers in bulk solution. δ,β 2D torsion free energy surface of a D-mannitol molecule in bulk solvent at 277K from accelerated Meta-dynamics MD simulations.

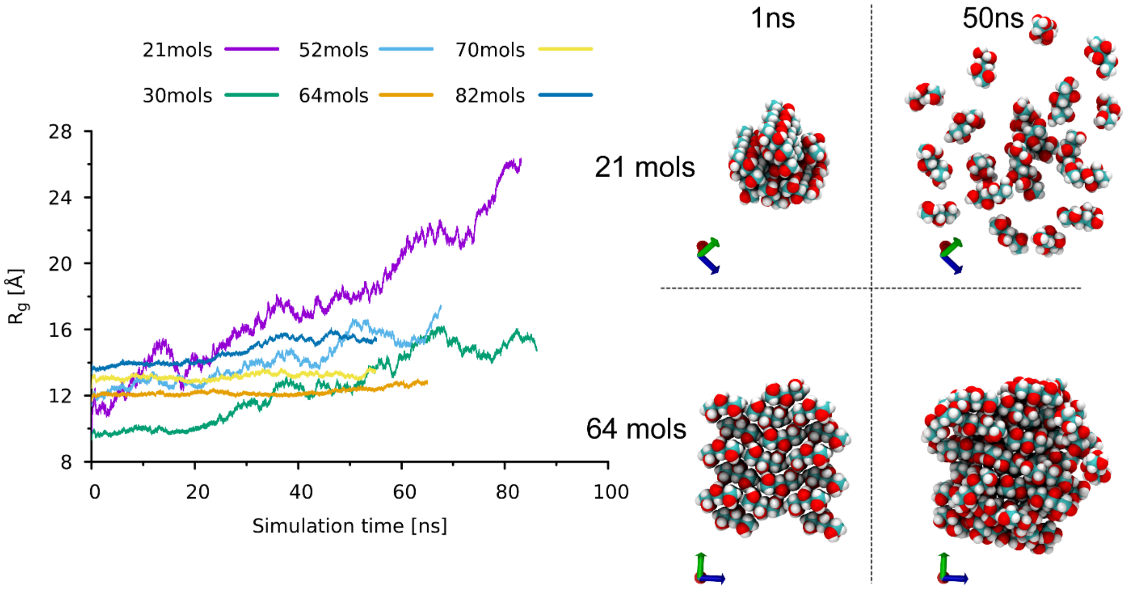

Figure S7. Stability of D-mannitol clusters. Time evolution of the radius of gyration (R_g) for various sized D-mannitol clusters in bulk solution, from unrestrained equilibrium MD simulations at 277K. We find that clusters with less than 64 molecules are mechanically unstable, disassociating on 100 ns time scales. Larger clusters (82), that exceed the critical nucleus size, are found to be stable. Snapshots of the system at the start (1ns) and end (50ns) of the simulation for the 21 and 64 molecule clusters are presented as a reference.

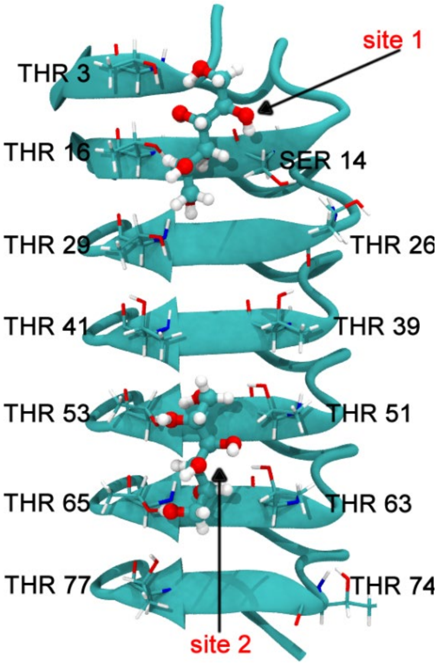

Figure S8. Low energy binding sites for D-mannitol on the IBS of DAFP1. Results are from the DarwinDock procedure, calculated in the absence of solvent molecules. We calculate ΔE_{bind} = -74.6 kJ/mol and -73.7 kJ/mol for site 1 and 2 respectively. The THR and SER residues on the IBS are also shown. D-mannitol makes strong hydrogen bonds with THR 16 and SER 14, and weaker interactions with THR 3 and THR 29 in site 1. In site 2, D-mannitol makes strong

hydrogen bonds with THR 53 and THR 65, and weaker interactions with THR 51, THR 63 and THR 77.

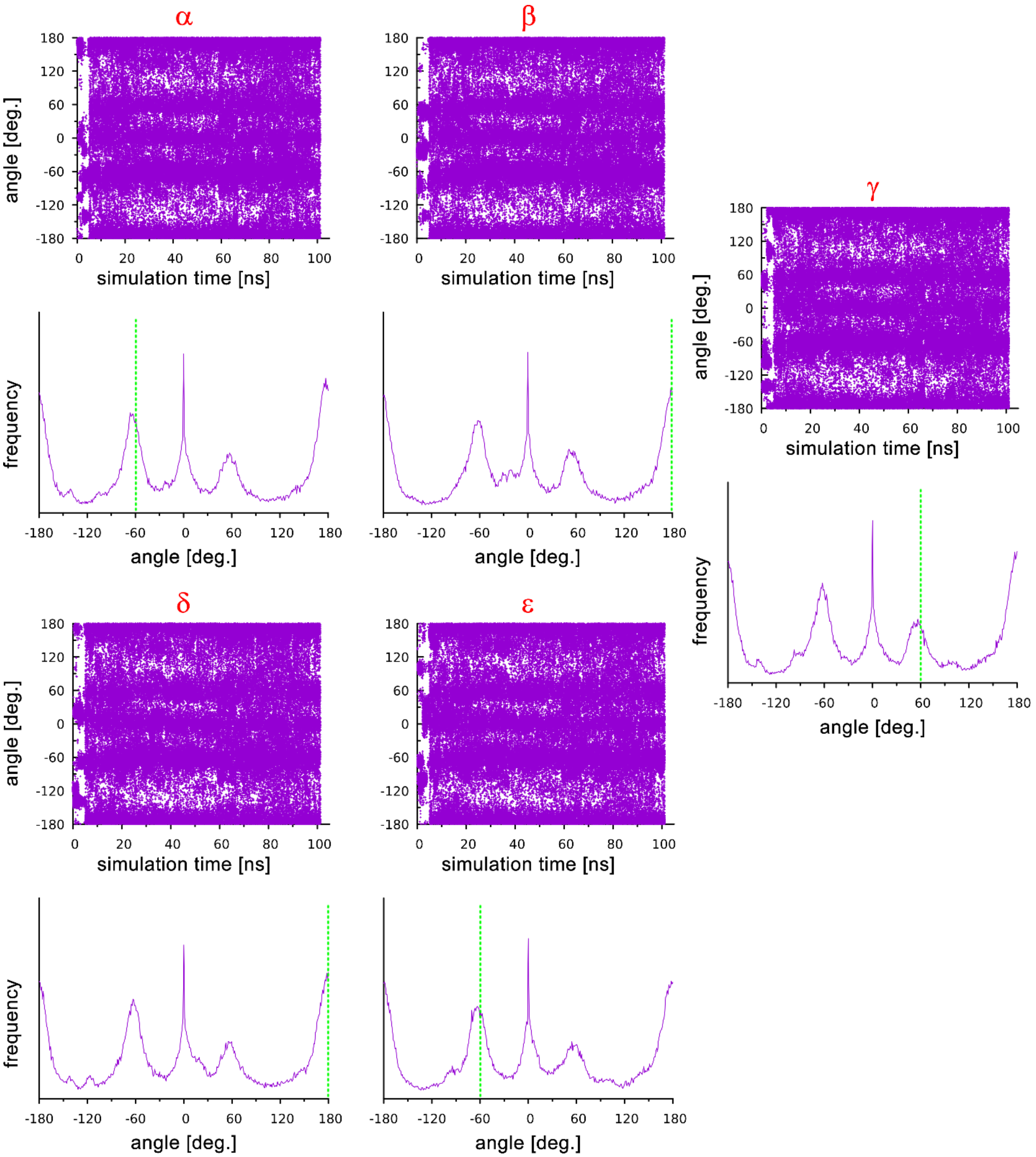

Figure S9. D-Mannitol torsional angles next to DAFP1. Time progressions of each of the 5 O-C-C-O torsions of D-mannitol, confined to the DAFP1 surface, from a 100 ns MD simulation, sampled every 10fs. The integrated probabilities for each angle are also shown. The dashed vertical lines in each frequency plot represents the average value of the crystal forming rotamer in solution. Note that in addition to +60° (+gauche), 180° (trans), and -60° (negative gauche), we find large populations at 0° (Cis) for all cases.

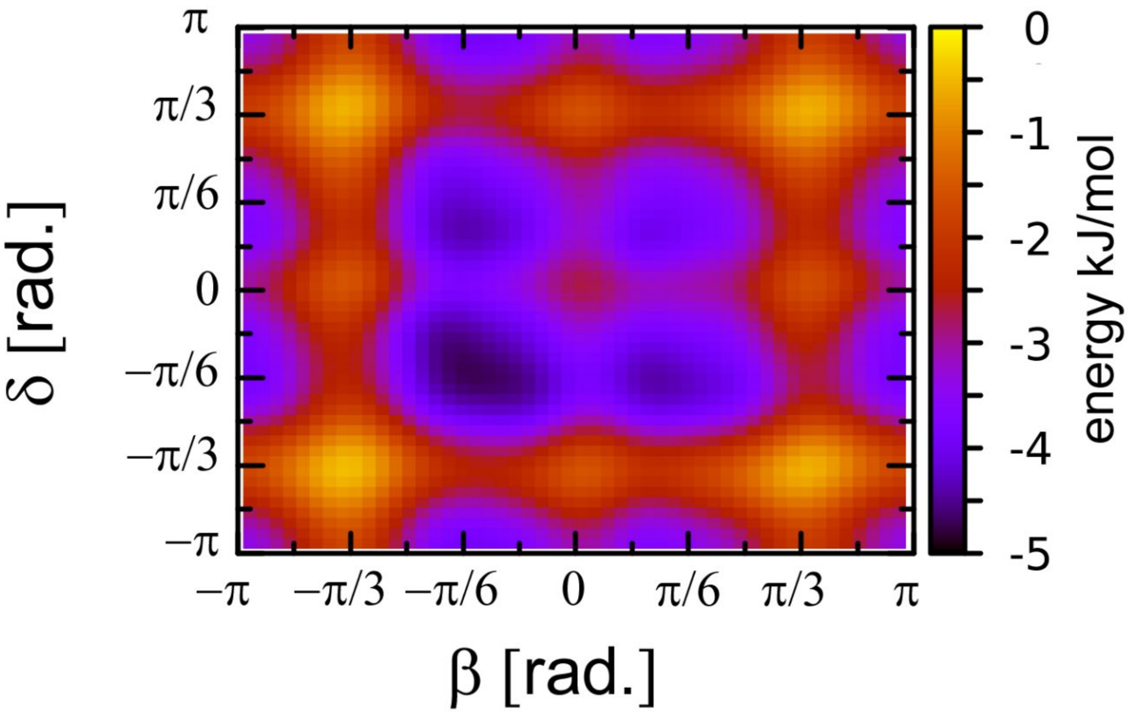

Figure S10. D-Mannitol torsional rotation barriers next to DAFP1. δ,β 2D torsion free energy surface of a D-mannitol molecule confined to the surface of DAFP1, at 277K from accelerated Meta-dynamics MD simulations. Note that the barriers (~ 3 - 4 kJ/mol) are comparable to kT, and are an order of magnitude less than the barriers in the bulk (**Figure S3**)

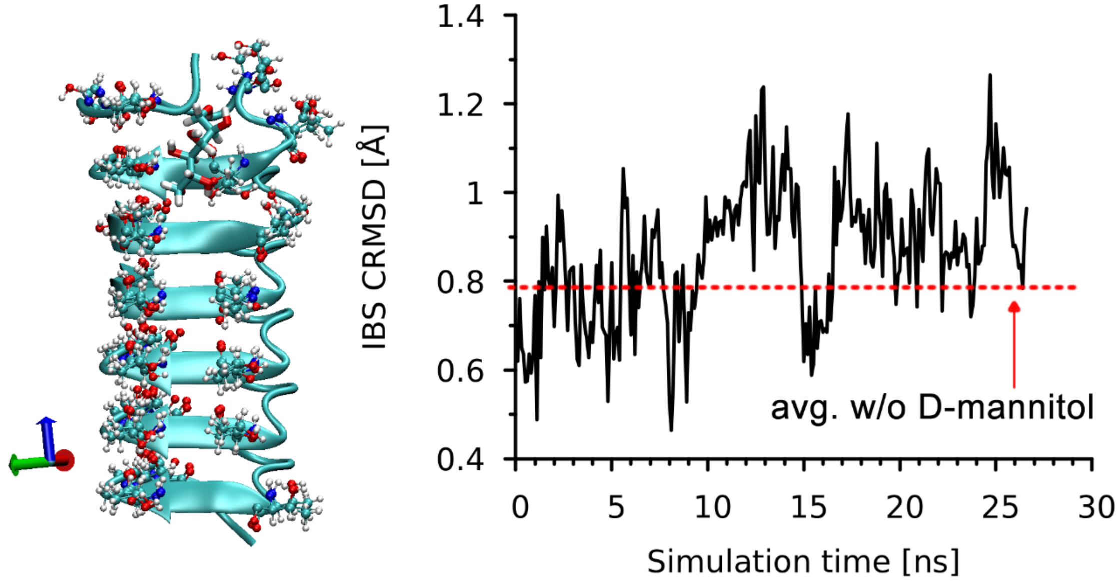

Figure S11. Structure of the DAFP-1 IBS with D-mannitol. Molecular structure (left) of DAFP-1, showing the SER and THR residues that comprise the IBS. Structure from the start, middle and end of the MD simulations are superimposed, showing that there is relatively little change during MD at 277K. This is quantified on the right by calculating the coordinate root mean square deviation (CRMSD) of these residues, using the starting MD structure as reference. This analysis shows a CRMSD of 0.9 ± 0.1 Å, which is comparable to the fluctuations in the IBS structure that we find from simulations without the bound D-mannitol (0.8 ± 0.1 Å)

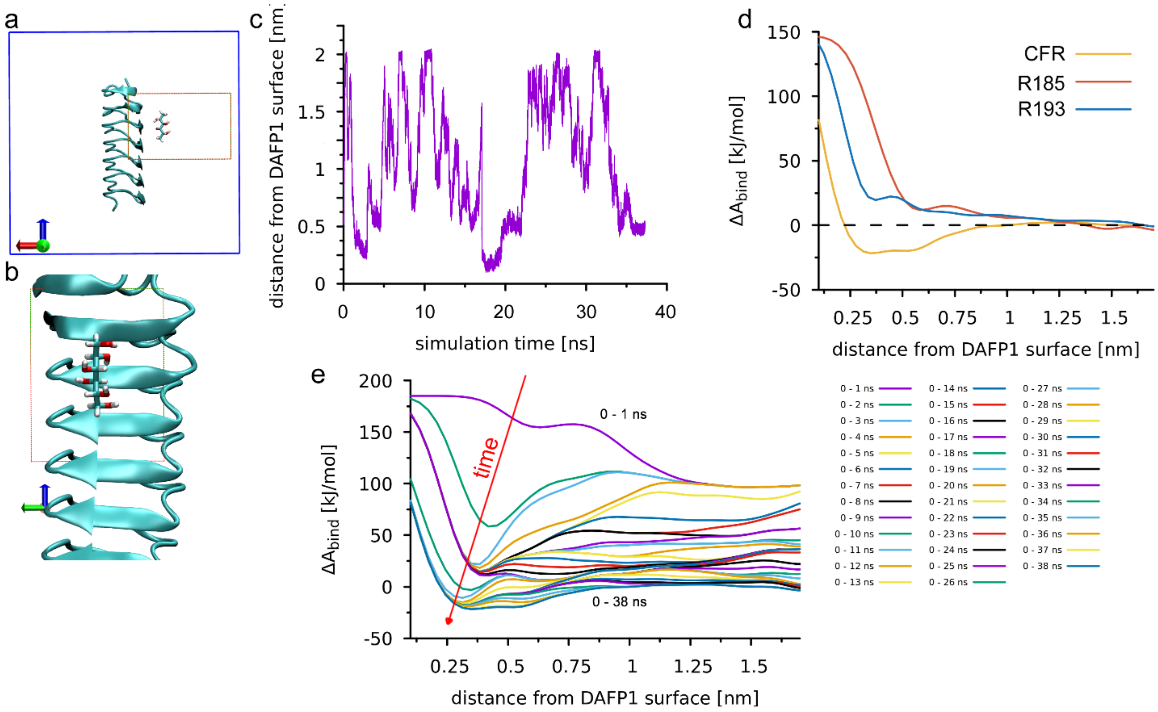

Figure S12. D-Mannitol binding thermodynamics to DAFP1. **a,** Schematic of DAFP1/D-mannitol simulation cell (solid blue line) used in accelerated Metadynamics free energy simulations. We restrict the D-mannitol within the dashed red box (site 1) for computational efficiency. **b,** Zoomed in of side-on view of crystallization rotamer next to DAFP1. The red-dashed box in a is shown. **c,** Time series of chosen collective variable, the X component of the center of mass position of D-mannitol, during Metadynamics. The center of mass of the DAFP1 molecule is restrained. **d,** Binding Gibbs energy curves for the crystal forming rotamer (CFR – yellow), most populous rotamer in the bulk solution (BR – green), and the two most populous rotamers on the DAFP1 surface: rotamer #185 (red) and rotamer #193 (blue). We restrain the rotamer internal structure throughout these simulation by application of rigid body dynamics. **e,** Convergence in the binding Gibbs energy of the NFR binding to site 1 on DAFP1. The running average of the free energy curve every 1ns of a 38ns Metadynamics simulation is presented. The red arrow line indicates increasing time.

Supplementary Tables

Table S1. D-mannitol crystal and nucleation. Sample results of D-mannitol crystal growth in the absence and presence of additives.

Samples[a]	Additive molar ratio	Induction time (day)[b]	Stop time (day)[c]	Weight of crystals (%)[d]
Mannitol	n/a	14 ± 0.3	200	82.4 ± 0.1%
Mannitol + GalNAc	1.0×10^{-2}	14 ± 0.3	200	82.5 ± 0.3%
Mannitol + denatured DAFP1	1.0×10^{-2}	14 ± 0.3	200	82.4 ± 0.2%
Mannitol + DAFP1	2.8×10^{-6}	n/a[e]	n/a[e]	n/a[e]
Mannitol + DAFP1	1.0×10^{-2}	n/a[e]	n/a[e]	n/a[e]
Mannitol + AFGP4-5	2.8×10^{-5}	61 ± 0.3	200	56.8 ± 0.2%
Mannitol + AFGP4-5	1.0×10^{-2}	63 ± 0.3	200	56.1 ± 0.3%
Mannitol + AFGP8	2.8×10^{-5}	41 ± 0.3	200	81.7 ± 0.1%

Mannitol + AFGP8	1.0×10^{-2}	41 ± 0.3	200	81.5 ± 0.2%

[a] Each sample contained 1.0 M mannitol on day 1. Results of the mannitol samples in the absence and presence of the controls, denatured DAFP1 and GalNAc, are listed for comparison. [b] The day that the first solid was observed. [c]The day no more weight increases for the solids in all the vials of D-mannitol in the absence of additives and the experiment was stopped. Time errors are 8 hours or less (i.e., ± 0.3 day). [d]The identity of the crystals were examined using polarized microscope and single crystal x-ray diffraction. [e]No crystallization of D-mannitol in the presence of DAFP1 was observed for more than 3 years.

Table S2. Crystallographic Data for D-Mannitol. Results are from control samples in the absence of any additives.

Formula	$C_6H_{14}O_6$
Formula Weight	182.17
Data collection temperature	100 K
Crystal system	orthorhombic
Space group	$P2_12_12_1$
Unit cell dimensions	
a, *b*, *c* (Å)	5.5347(4), 8.5755(6), 16.7801(12)
α, *β*, *γ* (°)	90°, 90°, 90°
Volume	796.43(10) Å^3
Z	4
Density (calculated)	1.519 g/cm^3
Reflections > 2σ(I)	5645
Average σ (I)/(net I)	0.0259
Data / restraints / parameters	1476 / 6 / 127
Final R indices [I>2σ(I)]	R_1 = 0.0253, wR_2 = 0.0669
R indices (all data)	R_1 = 0.0260, wR_2 = 0.0676

Table S3. Energies of all 243 rotamers in vacuum, bulk solution and on the surface of DAFP1. The relevant conformers: the crystal forming conformer (CFR), the most populous conformer in the bulk (BR) and the most likely conformers on the DAFP1 surface (#183 and #193) are indicated. Results from both Quantum Mechanical (QM) and Forcefield (FF) calculations are presented.

Conf #	O-C-C-O Torsion					Fractional Population (277K)					
	α	β	γ	δ	ε	QM(vacuum)	FF(vacuum)	FF(solvent)	<DAFP1>	±	
1	n	t	p	t	n	0.000677	0.001521	0.004512	0.0004	2.93E-05	*CFR
2	n	t	p	t	t	2.92E-05	0.001325	0.011582	0.000168	7.78E-06	
3	n	t	p	t	p	2.88E-05	0.01373	0.02422	0.000486	4.04E-05	
4	n	t	p	p	n	0.000182	2.03E-09	2.56E-06	0.001973	0.000104	
5	n	t	p	n	t	0.000142	1.7E-07	0.002401	0.00244	5.88E-05	
6	p	t	p	t	n	1.04E-06	2.95E-08	0.028306	0.000381	1.47E-05	
7	n	t	p	n	p	0.000118	5.09E-05	0.010463	0.001866	5.66E-05	
8	t	n	p	t	n	2.57E-05	6.28E-08	0.002195	0.00556	0.000186	
9	p	t	p	t	t	4.37E-06	6.78E-07	0.152193	0.000176	7.95E-06	
10	n	t	p	n	n	1.9E-14	9.2E-05	0.000269	0.00254	0.000148	
11	t	n	p	t	t	0.00013	7.62E-07	0.001393	0.001129	4.05E-05	
12	p	t	p	t	p	2.41E-06	0.000636	0.010001	0.000886	8.72E-05	
13	t	t	p	t	n	0.002268	0.000112	0.015785	0.0003	1.65E-05	

14	p	p	n	p	n	2.72E-08	3.79E-14	2.13E-12	0.000261	1.40E-05	
15	t	n	p	t	p	1.9E-14	1.78E-05	0.011182	0.001265	4.44E-05	
16	n	t	p	p	p	2.38E-06	4.03E-08	2.01E-07	0.000655	3.98E-05	
17	p	p	p	t	n	0.001118	1.41E-06	6.54E-07	0.002569	4.69E-05	
18	p	t	p	p	n	3.6E-07	7.86E-14	1.35E-06	0.00154	4.53E-05	
19	t	t	p	t	t	9.06E-05	0.00068	0.33703	0.000174	5.65E-06	*BR
20	p	t	p	n	n	5.19E-05	0.000532	0.017671	0.001854	5.31E-05	
21	p	t	p	n	t	7.83E-06	6.09E-06	0.002758	0.002268	7.64E-05	
22	t	t	p	t	p	1.43E-05	0.000168	0.116122	0.000418	4.29E-05	
23	p	p	p	t	t	0.000561	2.42E-06	0.005997	0.000796	3.72E-05	
24	p	t	p	n	p	3.27E-07	9.62E-05	0.001144	0.002693	7.90E-05	
25	p	n	p	t	n	7.76E-08	2.1E-09	0.006632	0.000376	2.86E-05	
26	t	p	n	p	n	2.53E-08	2.93E-19	4.96E-12	0.001143	3.26E-05	
27	p	p	p	t	p	5.32E-05	4.19E-06	3.39E-06	0.000684	3.82E-05	
28	p	n	p	t	t	9.85E-07	4.31E-09	0.005628	0.000442	3.74E-05	
29	n	p	p	t	n	5.86E-05	1.53E-07	1.01E-06	0.000818	1.45E-05	
30	t	n	p	n	n	0.067	0.000133	0.000189	0.008684	0.000512	
31	n	p	n	p	n	2.64E-09	3.37E-17	5.33E-12	0.000486	1.31E-05	
32	t	n	p	p	n	0.000408	1.08E-11	6.96E-08	0.00302	0.000124	
33	n	n	p	t	t	1.09E-05	4.31E-08	0.004652	0.001688	6.33E-05	
34	p	n	p	t	p	5.49E-07	1.76E-06	0.001378	0.000472	1.30E-05	
35	t	t	p	p	n	0.000274	2.38E-17	6.96E-06	0.000865	9.58E-05	
36	t	t	p	n	n	0.000271	0.000596	0.002373	0.000537	1.93E-05	
37	t	n	p	n	p	0.006706	0.000194	2.41E-06	0.00367	0.000178	
38	n	p	p	t	t	4.72E-06	1.95E-07	1.01E-06	0.000562	3.41E-05	
39	t	t	p	n	t	1.63E-05	2.74E-05	0.005565	0.000896	2.48E-05	
40	t	n	p	n	t	0.100058	2.24E-05	2.24E-05	0.00157	7.08E-05	
41	n	n	p	t	n	1.69E-05	2.03E-07	0.001272	0.003165	0.000114	

42	n	p	p	t	p	1.93E-06	2.1E-08	1.48E-06	0.002005	0.0001
43	t	t	p	n	p	1.8E-05	0.001077	0.005078	0.000971	2.85E-05
44	n	n	p	t	p	0.000266	0.001642	0.000167	0.003191	5.82E-05
45	p	p	p	p	n	0.000201	4.22E-12	4.08E-06	0.0017	7.23E-05
46	p	t	p	p	p	5.15E-11	1.12E-12	0.054291	0.000116	4.09E-06
47	p	p	p	n	n	0.000634	5.96E-09	4.06E-07	0.000516	1.31E-05
48	p	n	p	n	n	4.02E-05	9.68E-06	9.48E-05	0.000939	3.95E-05
49	n	n	p	n	n	0.198001	0.043995	4.4E-05	0.001319	4.37E-05
50	p	p	p	n	t	0.000285	7.18E-06	1.13E-07	0.000694	4.78E-05
51	p	p	n	p	p	6.49E-09	1.33E-15	1.43E-10	0.000124	7.90E-06
52	p	p	p	n	p	1.34E-07	3.43E-10	6.32E-08	0.000533	3.24E-05
53	n	t	t	t	t	0.000204	0.0003	8.38E-05	0.002249	6.58E-05
54	p	n	p	p	n	1.79E-07	5.62E-08	3.28E-07	0.000584	3.30E-05
55	t	n	p	p	p	0.000145	1.35E-06	0.000743	0.001012	5.78E-05
56	p	n	p	n	p	1.01E-05	2.85E-07	1.04E-05	0.00071	2.98E-05
57	n	n	p	n	t	0.003277	1.55E-05	4.28E-05	0.001563	9.77E-05
58	n	n	p	n	p	0.003286	0.001028	2.87E-05	0.002289	0.000194
59	p	n	p	n	t	4.01E-05	2.1E-06	4.47E-06	0.002236	9.77E-05
60	n	t	t	t	n	0.000329	7.54E-05	8.17E-06	0.003908	0.000114
61	n	n	p	p	n	0.005748	4.37E-09	3.78E-07	0.00402	0.000225
62	t	t	p	p	p	9.05E-08	2.07E-08	6.45E-07	0.00037	4.25E-05
63	n	p	p	n	n	3.5E-06	8.57E-07	2.84E-07	0.00061	3.65E-05
64	p	p	p	p	p	2.12E-06	4.5E-13	0.016782	0.001119	5.61E-05
65	n	p	p	p	n	2.96E-05	7.11E-14	3.6E-10	0.004512	0.000161
66	n	p	p	n	t	5.37E-08	5.64E-09	6.14E-07	0.003148	0.000134
67	t	p	n	p	p	3.94E-10	4.62E-18	4.94E-11	0.000619	2.79E-05
68	n	t	t	t	p	1.23E-05	0.001289	1.34E-05	0.003019	8.84E-05
69	n	p	n	p	p	4.28E-07	1.54E-18	6.16E-12	0.000475	2.87E-05

70	n	p	p	n	p	1.76E-07	4.56E-10	2.61E-07	0.000306	1.71E-05
71	p	t	t	t	t	0.000117	0.000356	6.24E-05	0.000412	1.13E-05
72	n	t	t	n	t	0.000199	1.47E-05	0.000135	0.000489	2.91E-05
73	p	t	t	t	n	0.00118	0.000866	1.22E-05	0.002035	0.000116
74	n	t	t	n	p	1.02E-06	0.000113	8.09E-05	0.000583	1.26E-05
75	p	n	p	p	p	1.95E-07	4.56E-07	5.18E-08	7.69E-05	8.74E-06
76	n	n	p	p	p	1.74E-05	1.22E-10	1.57E-08	0.00089	2.93E-05
77	n	p	p	p	p	2.02E-08	1.79E-13	5.61E-11	0.000964	0.000117
78	t	t	t	t	t	0.000284	3.16E-05	6.23E-05	0.00055	3.67E-05
79	t	t	t	t	n	0.000309	3E-05	8.76E-05	0.001017	4.25E-05
80	n	t	n	p	n	0.005313	3.76E-12	1.07E-09	0.002122	7.35E-05
81	p	t	t	t	p	3.19E-05	0.001712	1.81E-05	0.000749	2.32E-05
82	p	t	t	n	t	1.67E-06	1.84E-06	0.000498	0.000736	6.00E-05
83	p	t	t	n	p	6.55E-10	7.31E-09	0.000122	0.000406	7.43E-06
84	t	t	t	t	p	1.29E-05	0.000176	6.55E-05	0.000684	5.12E-05
85	p	t	n	p	n	0.020128	1.02E-07	4.48E-10	0.003752	7.41E-05
86	t	t	t	n	t	0.000748	4.46E-05	8.47E-05	0.002682	6.41E-05
87	p	p	n	t	t	1.37E-05	2.9E-09	1.07E-09	0.001243	3.12E-05
88	p	p	n	t	n	2.33E-06	5.25E-11	1.2E-09	0.001714	6.03E-05
89	t	t	t	n	p	3.35E-05	6.25E-05	6.19E-05	0.003059	6.21E-05
90	n	t	n	p	p	1.77E-06	1.18E-10	1.17E-09	0.001381	9.85E-05
91	t	n	t	t	n	0.000135	1.13E-09	0.000212	0.000435	2.60E-05
92	p	p	n	t	p	4.47E-06	2.69E-07	1.94E-09	0.001175	3.43E-05
93	t	t	n	p	n	0.008448	1.07E-10	5.28E-10	0.006207	0.000253
94	t	n	t	t	t	0.000372	1.35E-05	5.23E-05	0.000288	9.62E-06
95	p	p	n	p	t	9.62E-11	2.92E-17	3.62E-11	0.000956	7.98E-05
96	n	p	n	t	t	0.000793	1.4E-11	1.81E-10	0.001325	5.93E-05
97	t	n	t	t	p	1.72E-08	3.62E-09	3.96E-05	0.000755	2.86E-05

98	p	n	t	t	n	4.83E-10	4.17E-12	2.85E-05	0.003289	7.96E-05
99	n	p	n	t	n	0.000907	1.11E-12	2.01E-09	0.002695	7.86E-05
100	p	t	n	p	p	1.7E-05	4.62E-07	2.76E-10	0.001253	6.56E-05
101	n	p	n	t	p	0.002754	1.88E-08	7.68E-09	0.002419	7.47E-05
102	p	n	t	t	t	8.55E-10	4.31E-08	4.67E-05	0.001705	5.66E-05
103	n	p	n	p	t	3.69E-11	2.65E-19	9.42E-12	6.22E-05	5.16E-06
104	t	p	n	p	t	2.95E-09	5.67E-19	4.87E-11	0.001115	5.37E-05
105	p	n	t	t	p	2.03E-10	6.12E-11	0.000129	0.002201	5.34E-05
106	t	t	n	p	p	7.14E-06	1.4E-09	2.08E-09	0.003097	0.000139
107	t	p	n	t	t	9.5E-05	3.42E-15	4.36E-10	0.001008	4.87E-05
108	t	p	n	t	n	3.92E-07	5.02E-15	5.01E-10	0.001531	0.000103
109	t	p	n	t	p	0.000227	8.43E-13	9.91E-10	0.001074	4.93E-05
110	n	n	t	t	n	0.006434	9.69E-06	6.16E-06	0.001292	4.41E-05
111	n	n	t	t	t	0.002086	1.87E-05	8.63E-06	0.000732	4.06E-05
112	n	t	n	p	t	5.52E-06	4.1E-15	5.45E-10	0.000742	7.80E-05
113	n	n	t	t	p	0.061162	0.000137	4.91E-06	0.001126	5.61E-05
114	n	t	t	p	n	4.97E-05	3.45E-10	3.95E-09	0.000512	2.05E-05
115	n	t	p	p	t	3.81E-05	5.29E-11	7.44E-06	0.000522	2.53E-05
116	p	t	n	p	t	0.000515	7.03E-13	1.02E-09	0.002131	7.98E-05
117	t	p	p	t	n	0.00935	8.39E-07	8.69E-08	0.000112	6.52E-06
118	t	p	p	t	t	0.000249	2.39E-07	9.27E-06	6.79E-05	3.56E-06
119	p	t	t	p	n	2.27E-08	1.21E-09	4.75E-05	0.000437	5.46E-05
120	t	p	p	t	p	5.84E-05	8.43E-07	1.08E-06	0.000113	8.82E-06
121	t	t	n	p	t	2.85E-05	9.07E-16	3.79E-09	0.001541	4.16E-05
122	t	t	t	p	n	0.000325	9.54E-11	1.23E-08	0.001455	6.91E-05
123	t	p	p	p	n	0.000101	5.41E-12	7.47E-11	0.000998	3.80E-05
124	n	t	t	n	n	1.13E-06	5.17E-08	1.33E-06	0.000978	4.48E-05
125	p	t	p	p	t	2.37E-08	1.59E-16	0.070326	8.81E-05	4.54E-06

126	t	p	p	n	n	7.42E-06	1.62E-10	6.48E-08	0.000372	2.10E-05
127	t	p	p	n	t	1.25E-07	5.97E-12	0.004993	0.000358	2.04E-05
128	t	p	p	n	p	2.68E-07	2.03E-11	0.004479	0.00071	3.97E-05
129	t	n	p	p	t	0.001689	3.94E-06	0.000516	0.000666	2.10E-05
130	t	t	p	p	t	0.001628	7.23E-11	1.89E-06	0.000222	1.67E-05
131	p	t	t	n	n	5.24E-08	4.17E-08	3.27E-05	0.000863	2.65E-05
132	p	p	p	p	t	1.88E-06	1.71E-13	1.37E-07	0.000573	2.64E-05
133	t	p	p	p	p	1.13E-06	4.5E-12	4.49E-12	0.00106	7.18E-05
134	p	n	p	p	t	3.96E-06	9.84E-07	0.00582	2.22E-05	2.15E-06
135	n	p	p	p	t	2.18E-08	1.13E-15	8.28E-10	0.00061	5.63E-05
136	n	n	p	p	t	0.000597	4.67E-05	1.23E-07	0.002542	0.000149
137	t	t	t	n	n	0.016275	0.00075	3.13E-05	0.006689	0.00026
138	t	n	t	n	t	0.001764	5.88E-09	9.07E-06	0.000344	1.06E-05
139	n	t	t	p	p	6.11E-06	1.55E-07	3.51E-05	0.00022	8.90E-06
140	t	n	t	n	p	0.000198	9.12E-05	7.28E-05	0.000432	1.77E-05
141	n	p	t	t	t	2.58E-08	1.44E-09	4.8E-09	0.00066	2.92E-05
142	n	p	t	t	n	1.11E-09	2.9E-14	1.43E-09	0.001205	4.72E-05
143	p	n	t	n	t	2.53E-06	4.54E-08	0.000112	0.001363	7.26E-05
144	p	t	t	p	p	2.35E-15	1.14E-18	7.1E-06	4.04E-05	1.48E-06
145	p	n	t	n	p	6.27E-07	2.58E-06	0.000132	0.001291	4.02E-05
146	t	t	t	p	p	4.94E-06	1.15E-07	0.000232	0.001136	4.88E-05
147	n	p	t	t	p	2.14E-11	1.64E-12	6.73E-09	0.001642	5.74E-05
148	p	p	t	t	t	1.61E-08	2.26E-11	1.44E-05	0.000159	8.03E-06
149	p	p	t	t	n	6.1E-08	1.06E-11	7.26E-06	0.000321	2.17E-05
150	p	p	t	t	p	3.01E-09	2.31E-10	3.71E-05	0.000405	5.08E-05
151	n	n	t	n	t	0.012577	2.31E-05	9.72E-05	0.000305	1.51E-05
152	n	n	t	n	p	0.000716	0.00025	3.59E-05	0.001022	0.000127
153	p	n	n	p	n	1.31E-08	3.03E-15	9.54E-10	0.000819	4.58E-05

154	p	p	n	n	p	3.31E-12	1.1E-13	1.65E-09	0.000505	6.04E-05
155	n	p	n	n	p	4.71E-08	6.7E-16	2.05E-11	0.000552	2.74E-05
156	p	n	n	p	p	1.89E-11	6.67E-15	9.99E-11	0.000373	1.91E-05
157	t	p	n	n	p	5.28E-11	1.12E-15	9.79E-11	0.004955	0.000131
158	t	p	p	p	t	3.96E-06	8.5E-14	0.044173	0.000615	1.17E-05
159	t	n	n	p	n	5.07E-06	5.13E-13	8.49E-12	0.002533	0.00017
160	t	n	t	n	n	9.94E-05	4.05E-08	0.000824	0.00065	2.08E-05
161	t	n	n	p	p	3.49E-07	1.22E-10	4.06E-10	0.001961	0.000109
162	p	n	t	n	n	2.36E-06	6.01E-06	0.000151	0.000926	2.04E-05
163	p	n	n	p	t	2.27E-10	1.39E-16	9.61E-09	0.00013	3.62E-06
164	n	n	t	n	n	0.168115	0.925805	0.000375	0.000479	3.24E-05
165	t	n	n	p	t	4.05E-06	6.07E-14	1.57E-09	0.001557	9.67E-05
166	p	p	n	n	t	1.5E-07	5.27E-11	6.53E-10	0.001402	0.000112
167	n	p	n	n	t	1.29E-05	3.34E-12	1.54E-11	0.001609	6.00E-05
168	t	p	n	n	t	4.4E-06	2.46E-13	2.66E-10	0.001321	3.26E-05
169	n	t	n	t	t	0.13577	3.2E-09	4.62E-08	0.00035	3.71E-05
170	n	t	n	t	n	0.014454	5.07E-10	8.6E-10	0.000667	5.44E-05
171	n	t	n	t	p	0.11431	2.93E-05	1.67E-09	0.000584	7.05E-05
172	p	t	n	t	n	0.000791	2.07E-10	2.31E-09	0.000868	3.16E-05
173	p	t	n	t	t	0.005565	4.18E-06	1.33E-09	0.000311	1.23E-05
174	p	t	n	t	p	1.12E-05	3.06E-09	1.2E-09	0.000544	1.18E-05
175	t	t	n	t	t	0.000449	3.04E-09	2.23E-09	0.000342	1.21E-05
176	t	t	n	t	n	0.000116	4.27E-13	1.74E-08	0.000862	4.77E-05
177	t	t	n	t	p	0.000996	4.68E-07	2.25E-09	0.000631	5.34E-05
178	t	n	t	p	n	6.27E-05	9.02E-11	0.000131	0.000776	3.96E-05
179	p	n	t	p	n	7.12E-10	1.42E-15	0.000434	0.002274	6.25E-05
180	t	n	t	p	p	2.11E-09	7.11E-12	9.5E-05	0.000684	3.41E-05
181	p	n	t	p	p	1.77E-11	3.37E-12	0.00025	0.002224	0.000102

182	n	n	t	p	p	1.05E-08	1.14E-13	6.08E-06	0.001545	4.65E-05
183	n	n	t	p	n	2.39E-06	1.05E-10	2.56E-06	0.000958	4.42E-05
184	n	p	t	n	t	3.09E-09	1.01E-09	0.000219	0.001973	9.15E-05
185	n	p	t	n	p	4.35E-11	1.98E-09	5.18E-05	0.009889	0.000396
186	p	p	t	n	t	1.06E-10	3.44E-13	0.000423	0.002869	6.14E-05
187	t	p	t	n	p	1.17E-11	1.1E-11	0.000174	0	0
188	p	p	t	n	n	1.87E-09	1.03E-12	0.000132	0.001152	5.86E-05
189	n	p	t	n	n	2.59E-05	3.94E-10	2.07E-05	0.002276	9.31E-05
190	p	p	n	n	n	1.34E-06	1.32E-10	2.46E-12	0.000233	2.10E-05
191	n	p	n	n	n	1.71E-06	3.46E-12	3.48E-12	0.000611	5.87E-05
192	t	p	n	n	n	3.19E-05	5.01E-14	3.76E-12	0.001553	4.45E-05
193	n	n	n	p	n	0.000432	1.08E-10	4.56E-12	0.011925	0.000428
194	n	n	n	p	p	1.48E-06	2.8E-08	1.02E-12	0.006595	0.000216
195	n	n	n	p	t	4E-07	2.69E-15	9.2E-13	0.007739	0.00023
196	t	p	t	t	n	3.06E-08	6.42E-13	1.6E-08	0.000313	5.61E-05
197	t	p	t	t	p	2.25E-10	4.96E-12	6.83E-06	0.000256	2.07E-05
198	t	p	t	t	t	2.82E-09	2.77E-13	0.000778	9.96E-05	8.95E-06
199	n	t	n	n	t	2.6E-07	7.42E-13	2.76E-12	0.000373	1.25E-05
200	p	t	n	n	t	7.2E-13	8.49E-23	2.22E-11	0.002308	9.72E-05
201	t	t	n	n	t	2.64E-08	9.01E-14	1.05E-10	0.001054	6.85E-05
202	n	n	n	t	t	5.76E-05	3.61E-14	2.16E-12	0	0
203	t	n	n	t	n	1.48E-07	1.11E-19	4.97E-11	0	0
204	t	n	n	t	p	2.68E-09	6.82E-18	1.6E-10	0.001512	2.96E-05
205	n	t	n	n	p	2.07E-14	3.06E-20	7.68E-11	0.001209	9.99E-05
206	p	t	n	n	p	3.08E-09	5.83E-12	1.42E-11	0.00317	8.05E-05
207	t	t	n	n	p	2.75E-12	1.42E-19	2.53E-11	0.001344	3.94E-05
208	t	n	n	t	t	5E-13	4.41E-19	3.7E-11	0.000541	2.46E-05
209	t	n	n	t	n	5.57E-13	4.8E-20	3.77E-11	0.001102	2.75E-05
210	p	n	n	t	p	3.64E-13	1.1E-23	5.64E-11	0.000533	2.78E-05

211	n	t	t	p	t	1.51E-07	4.07E-10	1.56E-05	0.000161	1.28E-05
212	p	t	t	p	t	7E-13	2.07E-15	7.16E-06	6.17E-05	7.67E-06
213	t	t	t	p	t	8.31E-08	1.14E-10	1.15E-05	0.000652	4.59E-05
214	t	n	t	p	t	5.72E-08	2.97E-12	7.15E-05	0.000402	3.29E-05
215	p	n	t	p	t	4.11E-08	1.17E-17	0.00017	0.000908	2.87E-05
216	n	n	t	p	t	3.01E-09	2.71E-16	1.51E-06	0.00103	4.84E-05
217	t	n	n	n	t	5.68E-08	3.01E-17	1.5E-10	0.003189	7.17E-05
218	t	n	n	n	p	1.79E-10	5.2E-17	1.93E-10	0	0
219	p	n	n	n	t	7.46E-11	3.31E-19	1.08E-10	0.004878	0.000179
220	t	p	t	n	p	3.04E-08	1.37E-19	0.000409	0.000586	1.69E-05
221	t	p	t	n	t	9.52E-07	8.59E-12	0.000813	0.000602	2.54E-05
222	t	p	t	n	n	0.000499	1.35E-10	3.94E-05	0.000386	1.90E-05
223	p	n	n	n	p	7.38E-13	1.54E-19	7.22E-10	0.002834	0.000144
224	p	p	t	p	t	1.14E-14	3.98E-22	1.47E-05	0.000148	1.12E-05
225	n	p	t	p	t	5.34E-15	1.61E-27	4.47E-09	0.001365	8.57E-05
226	n	t	n	n	n	0.002814	3.14E-10	3.53E-11	0.000469	1.52E-05
227	p	t	n	n	n	2.37E-05	1.47E-10	3.83E-13	0.00341	0.000115
228	t	t	n	n	n	9.78E-06	3.13E-11	2.36E-12	0.000897	7.20E-06
229	p	p	t	p	p	2.08E-14	9.44E-20	1.29E-08	0	0
230	p	p	t	p	n	2.38E-11	3.78E-19	4.07E-09	0	0
231	t	p	t	p	p	2.83E-16	4.7E-22	0.000434	0.000183	1.30E-05
232	n	n	n	t	t	1.45E-05	1.51E-10	1.66E-12	0.000785	5.16E-05
233	n	n	n	t	n	0.004409	1.3E-09	3.51E-12	0.001251	5.87E-05
234	n	n	n	t	p	7.81E-10	6.66E-15	1.09E-11	0.001294	5.41E-05
235	t	n	n	n	p	1.5E-11	7.47E-24	5.8E-11	0.001795	0.000111
236	p	n	n	n	n	6.17E-15	6.7E-23	2.48E-11	0.003635	0.000135
237	n	n	n	n	t	1.17E-11	1.11E-24	8.82E-11	0.002814	0.000132
238	n	n	n	n	p	2.11E-11	8.91E-18	1.17E-10	0.002018	9.45E-05
239	n	n	n	n	n	2.94E-15	1.64E-28	5.59E-11	0.001319	0.000125

240	n	p	t	p	n	1.33E-12	4.82E-18	3.64E-09	0.002373	0.000103
241	p	p	t	p	n	1.41E-13	1.78E-20	2.95E-09	0.000235	8.97E-06
242	n	p	t	p	p	1.83E-12	4.45E-18	2.76E-09	0.00065	1.65E-05
243	p	p	t	p	p	3.74E-12	1.32E-18	0.000533	0.000112	4.02E-06

Table S4. Thermodynamic properties of bulk crystalline D-mannitol and the TIP4P-ice water model. Results are calculated from 277K equilibrium MD simulations with the Two-Phase Thermodynamics method. Experimental values are provided as a reference, where available.

	D-mannitol		water	
	Calc (This work)	Expt.	TIP4P-ice (calc – This Work)	Expt.
density ρ (g/cm^3)	1.42	1.52[a],1.49[b]	0.983	0.999
Dissolution energy (kJ/mol)	21.0 ± 0.5	22.6[c]		
Entropy S (J/mol/K)	213.6 ± 1.8	238.5, 253.1[d]	46.8	
Specific heat capacity C_p (J/mol/K)	220.0 ± 3.8	239.0[e]	71.2	75.7[f]
Interfacial surface tension σ (mJ/m^2)	34.4 ± 4.0	38 – 40[g]		

[a]This study, [b]Ref.[57], [c]Ref[58], [d]Refs[59,60], [e]Ref[61], [f]Ref[62], [g] at 298K, from Ref[63]

Table S5. D-mannitol binding to DAFP1 energies. Decomposition of D-mannitol rotamer binding to DAFP1 from the DarwinDock procedure.

CFR

Site	ΔE^{Total}	$\Delta E^{coulomb}$	$\Delta E^{h\text{-}bond}$	ΔE^{vdw}
1	-74.58	-14.29	-52.71	-7.58
2	-74.31	-13.50	-52.68	-8.11

BR

Site	ΔE^{Total}	$\Delta E^{coulomb}$	$\Delta E^{h\text{-}bond}$	ΔE^{vdw}
1	-43.99	-15.56	-17.54	-10.87
2	-42.16	-9.47	-28.07	-4.62

Table S6. Stability of D-mannitol rotamers: Normalized populations of various D-mannitol rotamers in the bulk solution before and after binding by DAFP1. Note that the best rotamer (BR) is predicted to not significantly bind to DAFP1, and so its population is relatively unaffected.

Rotamer	Bulk	±	After DAFP1	±
CFR	0.004512	0.0001	2.0E-06	1.0E-07
BR	0.33703	0.01	0.33602	0.013
R185	5.18E-05	1.00E-06	0.010877	0.003957
R193	4.56E-12	1.00E-13	0.013117	0.004277

Supplemental References

1. Fronczek, F.R., Kamel, H.N., and Slattery, M. (2003). Three polymorphs ([alpha], [beta], and [delta]) of d-mannitol at 100 K. Acta Crystallographica Section C *59*, o567-o570. doi:10.1107/S0108270103018961.
2. Griffith, A.R. (2017). DarwinDock and GAG-Dock: Methods and Applications for Small Molecule Docking. (California Institute of Technology).
3. Ewing, T.J., Makino, S., Skillman, A.G., and Kuntz, I.D. (2001). DOCK 4.0: search strategies for automated molecular docking of flexible molecule databases. Journal of computer-aided molecular design *15*, 411-428.
4. Mayo, S.L., Olafson, B.D., and Goddard, W.A. (1990). DREIDING: a generic force field for molecular simulations. J. Phys. Chem. *94*, 8897-8909.
5. Tak Kam, V.W., and Goddard III, W.A. (2008). Flat-bottom strategy for improved accuracy in protein side-chain placements. Journal of chemical theory and computation *4*, 2160-2169.
6. O'Boyle, N.M., Banck, M., James, C.A., Morley, C., Vandermeersch, T., and Hutchison, G.R. (2011). Open Babel: An open chemical toolbox. Journal of Cheminformatics *3*, 33. 10.1186/1758-2946-3-33.
7. Shao, Y., Gan, Z., Epifanovsky, E., Gilbert, A.T., Wormit, M., Kussmann, J., Lange, A.W., Behn, A., Deng, J., and Feng, X. (2015). Advances in molecular quantum chemistry contained in the Q-Chem 4 program package. Mol Phys *113*, 184-215.
8. Møller, C., and Plesset, M.S. (1934). Note on an approximation treatment for many-electron systems. Phys. Rev. *46*, 618.
9. Dunning Jr, T.H. (1989). Gaussian basis sets for use in correlated molecular calculations. I. The atoms boron through neon and hydrogen. J. Chem. Phys. *90*, 1007-1023.
10. Plimpton, S. (1995). FAST PARALLEL ALGORITHMS FOR SHORT-RANGE MOLECULAR-DYNAMICS. J. Comput. Phys. *117*, 1-19.
11. Guvench, O., Mallajosyula, S.S., Raman, E.P., Hatcher, E., Vanommeslaeghe, K., Foster, T.J., Jamison, F.W., and MacKerell, A.D. (2011). CHARMM Additive All-Atom Force Field for Carbohydrate Derivatives and Its Utility in Polysaccharide and Carbohydrate–Protein Modeling. Journal of Chemical Theory and Computation *7*, 3162-3180. 10.1021/ct200328p.
12. Kim, S., Lee, J., Jo, S., Brooks III, C.L., Lee, H.S., and Im, W. (2017). CHARMM-GUI ligand reader and modeler for CHARMM force field generation of small molecules. Wiley Online Library.
13. Jo, S., Kim, T., Iyer, V.G., and Im, W. (2008). CHARMM-GUI: a web-based graphical user interface for CHARMM. J Comput Chem *29*, 1859-1865.
14. Abascal, J.L.F., Sanz, E., Fernández, R.G., and Vega, C. (2005). A potential model for the study of ices and amorphous water: TIP4P/Ice. J. Chem. Phys. *122*, 234511. 10.1063/1.1931662.
15. Mochizuki, K., and Molinero, V. (2018). Antifreeze glycoproteins bind reversibly to ice via hydrophobic groups. J. Am. Chem. Soc. *140*, 4803-4811.
16. Wang, S., Amornwittawat, N., Juwita, V., Kao, Y., Duman, J.G., Pascal, T.A., Goddard, W.A., and Wen, X. (2009). Arginine, a Key Residue for the Enhancing Ability of an Antifreeze Protein of the Beetle Dendroides canadensis. Biochemistry *48*, 9696-9703.

17. Huang, J., Rauscher, S., Nawrocki, G., Ran, T., Feig, M., de Groot, B.L., Grubmüller, H., and MacKerell, A.D. (2017). CHARMM36m: an improved force field for folded and intrinsically disordered proteins. Nature Methods *14*, 71-73. 10.1038/nmeth.4067.
18. Mackerell Jr, A.D., Feig, M., and Brooks III, C.L. (2004). Extending the treatment of backbone energetics in protein force fields: Limitations of gas-phase quantum mechanics in reproducing protein conformational distributions in molecular dynamics simulations. J Comput Chem *25*, 1400-1415.
19. Jo, S., Cheng, X., Islam, S.M., Huang, L., Rui, H., Zhu, A., Lee, H.S., Qi, Y., Han, W., and Vanommeslaeghe, K. (2014). CHARMM-GUI PDB manipulator for advanced modeling and simulations of proteins containing nonstandard residues. Advances in protein chemistry and structural biology *96*, 235-265.
20. Luo, Y., and Roux, B. (2010). Simulation of osmotic pressure in concentrated aqueous salt solutions. J. Phys. Chem. Lett. *1*, 183-189.
21. Hockney, R.W., and Eastwood, J.W. (1988). Computer simulation using particles, 1st Edition (Taylor & Francis Group).
22. Jurrus, E., Engel, D., Star, K., Monson, K., Brandi, J., Felberg, L.E., Brookes, D.H., Wilson, L., Chen, J., and Liles, K. (2018). Improvements to the APBS biomolecular solvation software suite. Protein Science *27*, 112-128.
23. Pascal, T.A., Schwartz, C.P., Lawler, K.V., and Prendergast, D. (2019). The purported square ice in bilayer graphene is a nanoscale, monolayer object. J. Chem. Phys. *150*, 231101.
24. Shrestha, B.R., Pillai, S., Santana, A., Donaldson Jr, S.H., Pascal, T.A., and Mishra, H. (2019). Nuclear Quantum Effects in Hydrophobic Nanoconfinement. J. Phys. Chem. Lett. *10*, 5530-5535.
25. Pascal, T.A., Villaluenga, I., Wujcik, K.H., Devaux, D., Jiang, X., Wang, D.R., Balsara, N., and Prendergast, D. (2017). Liquid Sulfur Impregnation of Microporous Carbon Accelerated by Nanoscale Interfacial Effects. Nano Lett. *17*, 2517-2523.
26. Li, C., Ward, A.L., Doris, S.E., Pascal, T.A., Prendergast, D., and Helms, B.A. (2015). Polysulfide-Blocking Microporous Polymer Membrane Tailored for Hybrid Li-Sulfur Flow Batteries. Nano Lett. *15*, 5724-5729.
27. Pascal, T.A., and Goddard III, W.A. (2014). Interfacial Thermodynamics of Water and Six Other Liquid Solvents. J. Phys. Chem. B *118*, 5943-5956.
28. Jeon, J., Kim, H., Goddard III, W.A., Pascal, T.A., Lee, G.-I., and Kang, J.K. (2012). The role of confined water in ionic liquid electrolytes for dye-sensitized solar cells. J. Phys. Chem. Lett. *3*, 556-559.
29. Pascal, T.A., Goddard III, W.A., Maiti, P.K., and Vaidehi, N. (2012). Role of specific cations and water entropy on the stability of branched DNA motif structures. J. Phys. Chem. B *116*, 12159-12167.
30. Pascal, T.A., and Goddard III, W.A. (2012). Hydrophobic Segregation, Phase Transitions and the Anomalous Thermodynamics of Water/Methanol Mixtures. J. Phys. Chem. B *116*, 13905-13912.
31. Pascal, T.A., Schärf, D., Jung, Y., and Kühne, T.D. (2012). On the absolute thermodynamics of water from computer simulations: A comparison of first-

principles molecular dynamics, reactive and empirical force fields. J. Chem. Phys. *137*, 244507.
32. Pascal, T.A., Goddard, W.A., and Jung, Y. (2011). Entropy and the driving force for the filling of carbon nanotubes with water. Proc. Natl. Acad. Sci. U. S. A. *108*, 11794-11798.
33. Pascal, T.A., Lin, S.-T., and Goddard III, W.A. (2011). Thermodynamics of liquids: standard molar entropies and heat capacities of common solvents from 2PT molecular dynamics. Phys. Chem. Chem. Phys. *13*, 169-181.
34. Pascal, T.A., He, Y., Jiang, S., and Goddard III, W.A. (2011). Thermodynamics of Water Stabilization of Carboxybetaine Hydrogels from Molecular Dynamics Simulations. J. Phys. Chem. Lett. *2*, 1757-1760.
35. Pascal, T.A., Abrol, R., Mittal, R., Wang, Y., Prasadarao, N.V., and Goddard, W.A. (2010). Experimental Validation of the Predicted Binding Site of Escherichia coli K1 Outer Membrane Protein A to Human Brain Microvascular Endothelial Cells IDENTIFICATION OF CRITICAL MUTATIONS THAT PREVENT E. COLI MENINGITIS. Journal of Biological Chemistry *285*, 37753-37761.
36. Shinoda, W., Shiga, M., and Mikami, M. (2004). Rapid estimation of elastic constants by molecular dynamics simulation under constant stress. Physical Review B *69*, 134103.
37. Martyna, G.J., Tobias, D.J., and Klein, M.L. (1994). Constant pressure molecular dynamics algorithms. J. Chem. Phys. *101*, 4177-4189.
38. Parrinello, M., and Rahman, A. (1981). Polymorphic transitions in single crystals: A new molecular dynamics method. J. Appl. Phys. *52*, 7182-7190.
39. Tuckerman, M.E., Alejandre, J., López-Rendón, R., Jochim, A.L., and Martyna, G.J. (2006). A Liouville-operator derived measure-preserving integrator for molecular dynamics simulations in the isothermal–isobaric ensemble. J. Phys. A: Math. Gen. *39*, 5629.
40. Humphrey, W., Dalke, A., and Schulten, K. (1996). VMD: visual molecular dynamics. Journal of molecular graphics *14*, 33-38.
41. https://github.com/atlas-nano/2PT
42. Pannir Sivajothi, S.S., Lin, S.-T., and Maiti, P.K. (2018). Efficient computation of entropy and other thermodynamic properties for two-dimensional systems using two-phase thermodynamic model. J. Phys. Chem. B *123*, 180-193.
43. Sun, T., Xian, J., Zhang, H., Zhang, Z., and Zhang, Y. (2017). Two-phase thermodynamic model for computing entropies of liquids reanalyzed. J. Chem. Phys. *147*, 194505.
44. Lin, S.-T., Maiti, P.K., and Goddard, W.A. (2010). Two-Phase Thermodynamic Model for Efficient and Accurate Absolute Entropy of Water from Molecular Dynamics Simulations. J. Phys. Chem. B *114*, 8191-8198. 10.1021/jp103120q.
45. Lin, S.T., Blanco, M., and Goddard, W.A. (2003). The two-phase model for calculating thermodynamic properties of liquids from molecular dynamics: Validation for the phase diagram of Lennard-Jones fluids. J. Chem. Phys. *119*, 11792-11805.
46. Desjarlais, M.P. (2013). First-principles calculation of entropy for liquid metals. Phys. Rev. E *88*, 062145.

47. Laio, A., and Parrinello, M. (2002). Escaping free-energy minima. Proc. Natl. Acad. Sci. U. S. A. *99*, 12562-12566.
48. Barducci, A., Bonomi, M., and Parrinello, M. (2011). Metadynamics. WIREs Computational Molecular Science *1*, 826-843. https://doi.org/10.1002/wcms.31.
49. Ensing, B., De Vivo, M., Liu, Z., Moore, P., and Klein, M.L. (2006). Metadynamics as a Tool for Exploring Free Energy Landscapes of Chemical Reactions. Accounts of Chemical Research *39*, 73-81. 10.1021/ar040198i.
50. Barducci, A., Bussi, G., and Parrinello, M. (2008). Well-tempered metadynamics: a smoothly converging and tunable free-energy method. Phys. Rev. Lett. *100*, 020603.
51. Raiteri, P., Laio, A., Gervasio, F.L., Micheletti, C., and Parrinello, M. (2006). Efficient reconstruction of complex free energy landscapes by multiple walkers metadynamics. J. Phys. Chem. B *110*, 3533-3539.
52. Limongelli, V., Bonomi, M., and Parrinello, M. (2013). Funnel metadynamics as accurate binding free-energy method. Proc. Natl. Acad. Sci. U. S. A. *110*, 6358-6363.
53. Kamberaj, H., Low, R., and Neal, M. (2005). Time reversible and symplectic integrators for molecular dynamics simulations of rigid molecules. J. Chem. Phys. *122*, 224114.
54. Bonomi, M., Bussi, G., Camilloni, C., Tribello, G.A., Banáš, P., Barducci, A., Bernetti, M., Bolhuis, P.G., Bottaro, S., and Branduardi, D. (2019). Promoting transparency and reproducibility in enhanced molecular simulations. Nature methods *16*, 670-673.
55. Tribello, G.A., Bonomi, M., Branduardi, D., Camilloni, C., and Bussi, G. (2014). PLUMED 2: New feathers for an old bird. Comput Phys Commun *185*, 604-613.
56. Bonomi, M., Branduardi, D., Bussi, G., Camilloni, C., Provasi, D., Raiteri, P., Donadio, D., Marinelli, F., Pietrucci, F., and Broglia, R.A. (2009). PLUMED: A portable plugin for free-energy calculations with molecular dynamics. Comput Phys Commun *180*, 1961-1972.
57. Burger, A., Henck, J.-O., Hetz, S., Rollinger, J.M., Weissnicht, A.A., and Stöttner, H. (2000). Energy/temperature diagram and compression behavior of the polymorphs of D-mannitol. Journal of pharmaceutical sciences *89*, 457-468.
58. Parks, G.S., and Manchester, K.E. (1952). The Heats of Solution of Erythritol, Mannitol and Dulcitol; Combustion Values for Liquid Polyhydroxy Alcohols. J. Am. Chem. Soc. *74*, 3435-3436.
59. Parks, G.S., Kelley, K.K., and Huffman, H.M. (1929). Thermal data on organic compounds. V. A revision of the entropies and free energies of nineteen organic compounds. J. Am. Chem. Soc. *51*, 1969-1973.
60. Parks, G.S., and Anderson, C.T. (1926). Thermal data on organic compounds. III. The heat capacities, entropies and free energies of tertiary butyl alcohol, mannitol, erythritol and normal butyric acid. J. Am. Chem. Soc. *48*, 1506-1512.
61. Hernández-Segura, G.O., Campos, M., Costas, M., and Torres, L.A. (2009). Temperature dependence of the heat capacities in the solid state of 18 mono-, di-, and poly-saccharides. The Journal of Chemical Thermodynamics *41*, 17-20.

62. Archer, D.G., and Carter, R.W. (2000). Thermodynamic Properties of the NaCl + H2O System. 4. Heat Capacities of H2O and NaCl(aq) in Cold-Stable and Supercooled States. J. Phys. Chem. B *104*, 8563-8584. 10.1021/jp0003914.
63. Cares-Pacheco, M., Vaca-Medina, G., Calvet, R., Espitalier, F., Letourneau, J.J., Rouilly, A., and Rodier, E. (2014). Physicochemical characterization of d-mannitol polymorphs: The challenging surface energy determination by inverse gas chromatography in the infinite dilution region. International journal of pharmaceutics *475*, 69-81.
